# Community-based mental health screening & referral for flood-affected women in rural Pakistan: an intervention feasibility study protocol

**DOI:** 10.1136/bmjopen-2025-104759

**Published:** 2025-10-23

**Authors:** Jai K Das, Michelle F Gaffey, Zoya Navid Ansari, Mushtaq Mirani, Farhana Tabassum, Maira Niaz, Amna Siddiqui, Fauziah Rabbani, Arjumand Rizvi, Imran Ahmed, Murad Khan, Zulfiqar Ahmed Bhutta

**Affiliations:** 1 Institute for Global Health & Development, The Aga Khan University, Karachi, Pakistan; 2 Department of Paediatrics & Child Health, The Aga Khan University, Karachi, Pakistan; 3 Centre for Global Child Health, The Hospital for Sick Children, Toronto, Ontario, Canada; 4 Brain & Mind Institute, The Aga Khan University, Karachi, Pakistan; 5 Community Health Sciences, The Aga Khan University, Karachi, Pakistan

**Keywords:** MENTAL HEALTH, Anxiety disorders, Depression & mood disorders, Community Participation, Feasibility Studies, Climate Change

## Abstract

**Introduction:**

South Asia carries the burden of a rapidly changing climate with floods and extreme heat. These disasters further translate into mental health distress, financial stress and detrimental effects on well-being, with women being the most vulnerable. This study aims to demonstrate that mental health screening, referral and resilience-building group sessions can be successfully administered by community health workers and primary health facility staff in a flood-affected rural population of women in Pakistan and provide evidence on the effectiveness of this approach for improving their mental health status.

**Methods and analysis:**

A quasi-experimental design with a comparison group will be used for the study, preceded by a formative phase. The formative phase evaluated the feasibility of mental health screening by Lady Health Workers (LHWs) in flood-affected areas using a qualitative approach such as focus group discussions and in-depth interviews. Manuals developed by the study team of mPareshan will be used to train LHWs, Lady Health Supervisors (LHS) and health facility staff. Following this, LHWs will briefly screen women aged 18 to 49 years, administer awareness-raising and resilience-building sessions and refer women who screen positive for depression or anxiety to a primary health facility. Physicians at the health facility will confirm the diagnosis and provide counselling to mild-moderate cases, while severe cases would be referred to specialists. Statistical evaluation of quantitative data and thematic content analysis of qualitative data will be conducted to assess the feasibility and impact of the intervention. This trial is registered at clinicaltrials.gov with number NCT06756165.

**Ethics and dissemination:**

The study acquired ethical approval from the Ethical Review Committee at Aga Khan University (2024-10475-30776) and the National Bioethics Committee (4-87/NBC-1158/23/481) in Islamabad. Approval was obtained from relevant provincial authorities. The trial will adhere to the ethical principles of autonomy, anonymity, confidentiality, equity and respect. All eligible participants will be provided with informed consent, details regarding the purpose and procedure of the study, and the right to withdraw at any time. Data and information will be anonymised and stored securely. Dissemination of the results of the trial will occur after its completion to stakeholders, participants and the public.

**Trial registration number:**

NCT06756165.

**STRENGTHS AND LIMITATIONS OF THIS STUDY**

- There is an existing gap, and interventions have not been evaluated to cater to the mental health needs of women through a primary health system in a developing country context.
- The design of the trial is unique and has not been previously evaluated in a flood-affected rural setting.
- The methodology allows for evaluating the feasibility and cost-effectiveness of providing mental health awareness and resilience-building services in a community setting.
- The trial focuses on training community health workers for easier social integration with women in the setting.
- Results of the trial once published can inform policy in low and middle-income country contexts.

## Background

 Climate change has emerged as an alarming crisis in recent years with natural disasters escalating across the globe.^1^ Large-scale flooding, cyclones and heatwaves are just a few of the climate change-related disasters that threaten health infrastructure, resources for survival and shelter. The scarcity of basic needs increases physiological and mental health issues within disaster and flood-affected areas.^2^,^6^ According to WHO, an estimated 250 000 additional deaths related to climate change-induced malnutrition, diarrhoea, malaria and heat stress are predicted to occur per year between 2030 and 2050.^1^ Similarly, 150 000 lives have been lost every year for the past 30 years because of the detrimental effects of climate change.^6^ In addition to increasing mortality, disasters cause mental health distress, financial stress and other detrimental effects on well-being.^2^

South Asia carries a high burden of the rapidly changing climate, especially involving floods and extreme heat, with approximately 750 million people, half of the population across eight countries, experiencing at least one climate-related disaster in the past two decades.^4 7^ These communities have struggled due to the devastation of their physical and economic assets accompanied by psychological distress. Recurrent flooding and a lack of well-developed mental health services have worsened the living conditions of South Asians.^8 9^ In June 2022, Pakistan experienced extreme flooding causing immense destruction of crops, houses and health infrastructure,^10 11^ with a lack of clean drinking water, no access to basic services and the displacement of over 15 million people.^10^ Accompanied by political instability and stigma towards mental health, and absence of the integration of mental health services in primary healthcare, more than 13 000 individuals in Pakistan have been severely mentally and physically affected as a consequence of these floods.^9 11^

Moreover, vulnerable populations such as women, children and economically disadvantaged individuals are at a higher risk of adverse outcomes due to climate change-related disasters.^2 3^ It is essential that those disproportionately affected by these disasters are urgently catered to. In the rural areas of Sindh province, women and children experience higher rates of common mental health disorders (CMDs), gender-based violence and stress.^10 12^ The fear of potential calamities within individuals may serve as a predisposition to mental health conditions such as post-traumatic stress disorder 0r recurrent anxiety attacks.^11^ Screening and management of CMDs has still not been included in the primary healthcare programmes currently available to most women and children in rural Pakistan. Approximately 56% of Sindh lacks the availability of psychiatrists, sufficient mental health facilities and funding, and effective legislation.^10 13^ While the Sindh government has developed a limited curriculum to train Lady Health Workers (LHWs) about CMDs, it does not include community-based climate change and mental health resilience-building activities. Therefore, it becomes necessary to combat women’s psychosocial problems by incorporating sessions focused on community-based climate change and mental health resilience-building.

The traumatic impact of unforeseen displacement, loss of loved ones, destruction of agricultural livelihoods and absence of basic health services could be addressed, at least in part, through a feasible and effective community-based mental health programme. Moreover, community approaches to building climate resilience are part of Pakistan’s national climate change policy,^14^ further underscored by the recent massive floods. By catering to the mental health and flood-related issues of women in rural areas of Sindh through an existing primary health system, there may be hope for timely identification and delivery of appropriate intervention. This study aims to demonstrate that mental health screening, referral services and resilience-building group sessions can be successfully administered by community health workers and the primary health system within a flood-affected rural area of Pakistan and assess its effectiveness.

## Conceptual framework

**Figure 1:**
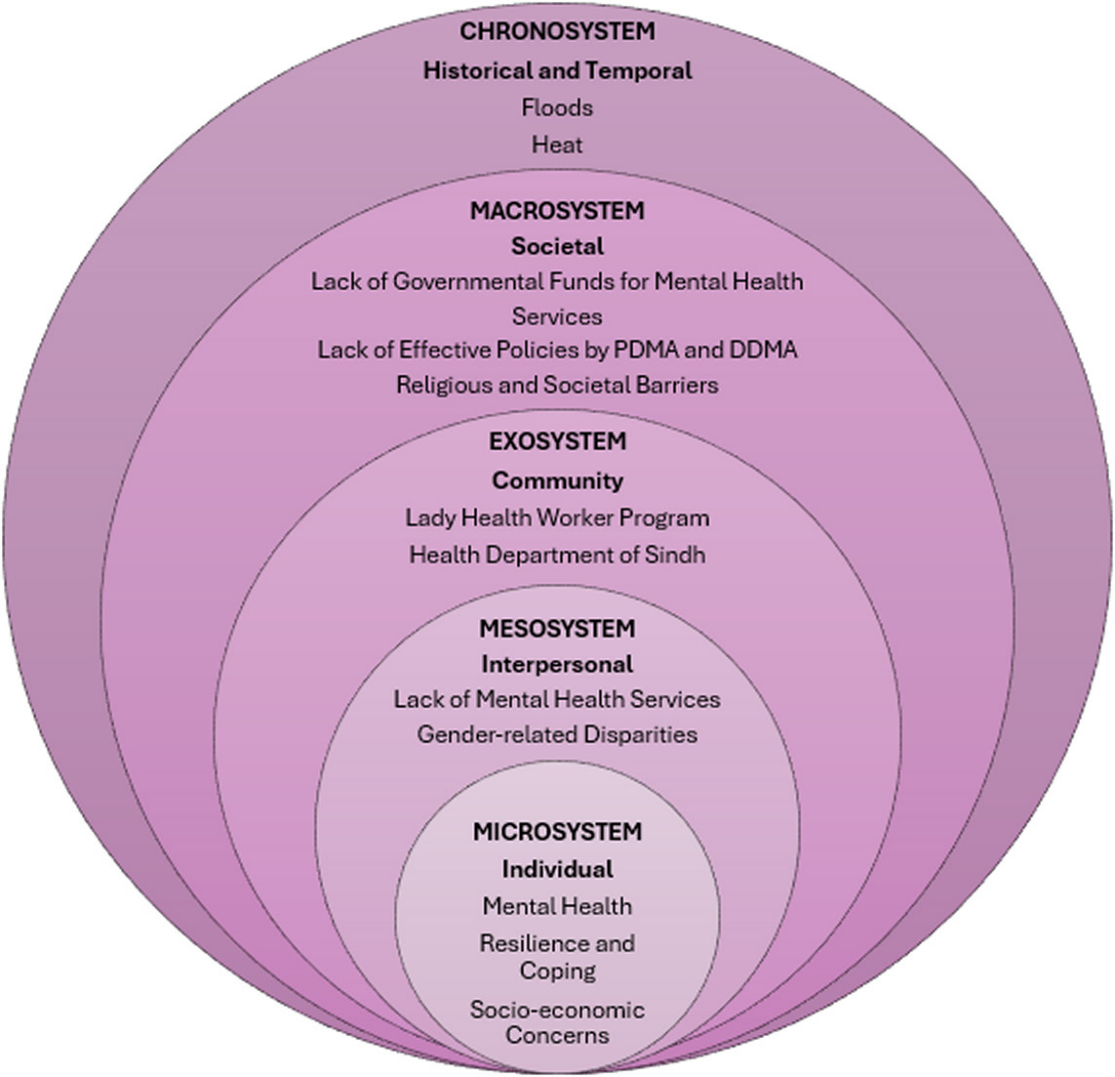
Conceptual framework based on the ecological systems theory by Bronfenbrenner. PDMA, Pakistan Disaster Management Authority; DDMA, District Disaster Management Authority.

The conceptual framework followed for the study was Bronfenbrenner’s ecological systems theory.^15^ It highlights the multifaceted levels of the healthcare system in relation to providing care to community members through effective delivery strategies, as depicted in figure 1. The microsystem works at an individual level involving issues regarding mental health, resilience and coping, and socioeconomic factors. The mesosystem further incorporates the lack of mental health services and gender-related disparities faced by the women of the community. At the community level, the exosystem focuses on providing services to the members through the LHW programme and the health department of Sindh. This level is essential to the research as the intervention focuses on evaluating the feasibility of integrating mental health services in LHW programme and government health facilities. The macrosystem working on a societal level provides clarity on the lack of governmental funds and effective policies by Pakistan Disaster Management Authority (PDMA), and the nuanced religious and societal barriers faced by women living in such communities which deter them from receiving and accessing quality healthcare. The chronosystem becomes significant as the natural disasters occurring in the region have worsened with time and combined with poverty and helplessness have led to the development of various mental health issues within our target population.

## Methodology and analysis

### Objectives

The primary objective of the study is to assess the feasibility, acceptability and appropriateness of implementing a community-based mental health screening, referral and resilience-building intervention for adult women of reproductive age (WRA) in a flood-affected rural community of the district of Dadu in Sindh, Pakistan. The secondary objective is to assess the effectiveness of this community-based intervention.

### Study design

A quasi-experimental design with a comparison group will be used for the study. The preceding formative work investigated the knowledge, attitudes and practices regarding mental health issues and resilience-building in the population, and the likely acceptability, appropriateness and feasibility of the planned screening and referral intervention using focus group discussions (FGDs) and in-depth interviews (IDIs). Findings from the formative work were then incorporated into the development of the training materials for LHWs and health facility staff.

To evaluate the feasibility and effectiveness of the screening and referral intervention, LHWs will be trained to screen WRA through the brief two-item Generalized Anxiety Disorder scale (GAD-2)^16^ and brief two-item Patient Health Questionnaire (PHQ-2),^17^ and administer awareness-raising and resilience-building sessions along with appropriate referral to Basic Health Unit (BHU). Facility staff present within BHU will be trained to confirm the screening using the 7-item Generalized Anxiety Disorder scale (GAD-7)^18^ and the 9-item Patient Health Questionnaire (PHQ-9),^19^ provide counselling and refer severe cases to specialists at tertiary care facilities.

The study duration is January to December 2025 with the initial few months focusing on the formative phase followed by the actual intervention. This trial is registered at clinicaltrials.gov with number NCT06756165.

### Study site

This study will be conducted in the flood-affected areas of Dadu district in Sindh province, Pakistan. Dadu is located west of the Indus River, approximately 160 km north-northwest of Hyderabad. It was one of the worst-affected areas during the flooding in 2022, with an immense increase in water-borne illnesses and loss of shelter, basic resources and agricultural livelihoods.^20^

The district of Dadu is administratively subdivided into four Talukas: Dadu, Johi, Khairpur Nathan Shah and Mehar. The study will take place specifically in Taluka Khairpur Nathan Shah, where 8 flood-affected Union Councils (UCs) were purposely chosen as they were the most severely affected and assigned into two groups: 4 UCs for the intervention group (Gozo, Burira, Chor Qambar and Kande-Chukhi) located on the left side of the Taluka Headquarters and 4 UCs for the control group (Dhani Bux Bughio, Parya, Thalo and Sindhi Butera) located on the right side to maintain distance and avoid contamination. Each UC in both arms has one BHU and a 24/7 Maternal, Newborn and Child Health (MNCH) Center.

### Population

The target population for this survey will be WRA within the ages of 18 years to 49 years. This sample was selected due to its critical relevance to the study’s focus on mental health and the impact of climate-related disasters, particularly flooding, on vulnerable individuals. Women in this age range are often at heightened vulnerability, balancing the physical demands of reproductive health with the psychological stressors posed by displacement and environmental crises. Based on the formative work preceding this study, women experienced traumatic events including sexual violence, rape, as well as a lack of privacy and security. Maternal health was severely affected as many pregnant women faced severe complications, infections and death.

### Sample Size

The sample size was calculated based on an estimated anxiety prevalence of 25.6%. To detect a 20% relative difference in prevalence between study arms with 90% power and a 5% level of significance, estimated intracluster correlation coefficient (ICC) of 0.0033 and an anticipated attrition rate of 10%, a total of 20 clusters per arm with 105 women per cluster (ie, 2100 women per arm) will be required. The prevalence of anxiety is assumed to vary by ±5 percentage points between clusters. The coefficient of variation (CV) was estimated based on this range, and the ICC was then derived using the CV formula.^21 22^

### Outcomes

**Figure 2:**
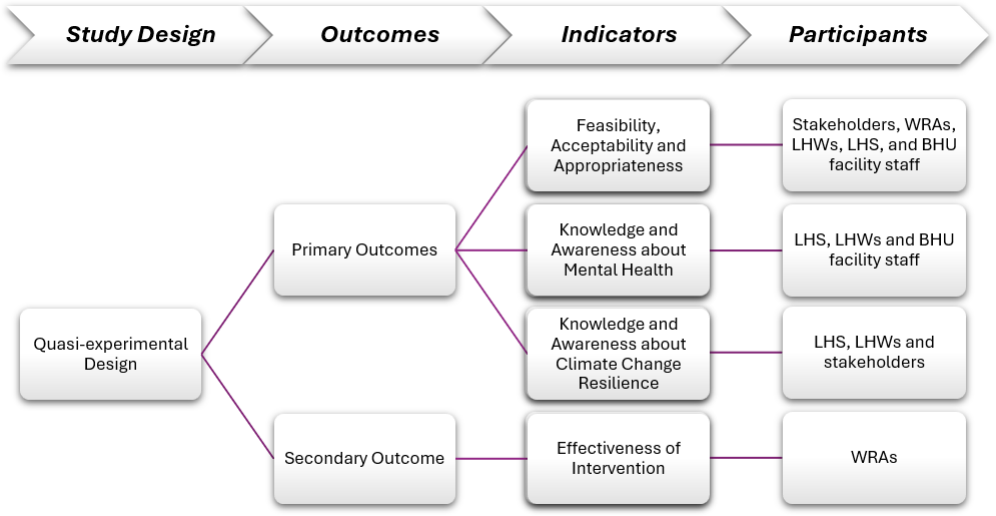
Study design and outcomes. BHU, Basic Health Unit; LHS, Lady Health Supervisors; LHWs, Lady Health Workers; WRA, Women of Reproductive Age.

The primary outcomes of the study include (figure 2):

- Feasibility, acceptability and appropriateness—assessed by proportion of WRA enrolled, proportion of screen-positive WRA (SP-WRA), determined by a cut-off score of 3 or greater,^16 17^ proportion of WRA attending mental health awareness and resilience-building group sessions conducted by LHWs, proportion of SP-WRA presenting to BHU facility for further assessment, proportion of SP-WRA diagnosed with mild-moderate anxiety and/or depression and receiving two counselling sessions by BHU staff at facility and proportion of WRA with severe depression and/or anxiety accepting referral to specialist.

The secondary outcome includes:

- Effectiveness of intervention on mental health—defined by the prevalence of anxiety and depression among WRA in the intervention arm when compared with the control arm.

### Formative phase

A qualitative formative study was conducted prior to the intervention phase to understand the dynamics influencing mental health and climate resilience in the target population. FGDs and IDIs were used to gather insights into community perceptions and assess the feasibility and relevance of the intervention from the perspective of various stakeholders (guides attached in online supplemental material). Verbal informed consent was acquired from all participants before data collection. This component assessed the feasibility and acceptability of implementing four key strategies: home-based mental health screening, referrals, group mental health awareness sessions and climate change-related resilience-building sessions, all facilitated by LHWs for WRA. Four IDIs were conducted with stakeholders including Disaster Management Officials and healthcare facility staff along with five FGDs with LHWs, Lady Health Supervisors (LHS), BHU facility doctors and community members.

The findings showed that many WRA did not understand the term ‘mental health’; hence, the intervention included sessions on mental health awareness to increase their knowledge about common mental health disorders such as depression and anxiety. Moreover, the stigma regarding mental health, for those who were aware, can be challenged through mental health and resilience-building sessions and counselling provided by healthcare facility staff at BHUs. WRA showed willingness for home-based mental health screenings and group sessions by LHWs, whom they perceived as trustworthy. Consequently, these interactions provided valuable insights into participants’ perceptions, existing knowledge of mental health and climate change, the preparedness of health facilities to handle referrals, and the capacity of LHWs to carry out screenings effectively which were incorporated into the intervention phase (table 1).

**Table 1:** Key findings of formative study (qualitative component)

Flood challenges and impacts	Participants unanimously regarded the 2022 flash floods as an unmitigated disaster, wiping out communities that received no prior warnings to evacuate. Many participants also lamented the rehabilitation process, with the entire experience leaving them physically drained and mentally traumatised. Women faced severe menstrual hygiene challenges due to a lack of menstrual products and a lack of privacy, using makeshift materials like dupattas. Sexual violence was also reported in shelters, especially in tents where families lived in close quarters, with minimal security. Some girls were raped, and victims’ families chose to remain silent due to social stigmas. Pregnant women suffered significantly, with some experiencing miscarriages and stillbirths due to stress, lack of medical care, and unsafe living and delivery conditions during the floods. Limited access to healthcare during the floods left pregnant women vulnerable to severe complications, including infections and maternal death.
Understanding of mental health	Initially, WRA did not understand the term ‘mental health,’ responding with confusion and silence. The trauma of the floods led to long-lasting mental health issues, with survivors experiencing symptoms like palpitations, anxiety and flashbacks during subsequent rains. Women, especially those who lost loved ones or their homes, were deeply affected, with some reporting persistent feelings of sadness, fear and helplessness. While there was recognition from almost all participants about the need for mental health services, some community members were unable to properly identify symptoms and issues.
Intervention feasibility and challenges	Overall, the proposed intervention was wholeheartedly welcomed by all study participants. Women highlighted challenges such as familial interference and refusals over permission to travel to healthcare facilities primarily and financial constraints. The stigma surrounding mental health in these communities was also highlighted as a challenge, with some women afraid of being labelled crazy and ostracised by others. While some facility staff questioned LHWs’ capabilities, community members highly trusted them, especially for maintaining privacy in sensitive areas like family planning and mental health. LHWs were also valued for their support in family planning and vaccinations. Facility staff highlighted issues related to space, training and human resources in their facilities, hindering the delivery of mental health support services. They also felt that issues with the existing LHW referrals system and record-keeping can cause logistical issues, leading to issues with tracking and follow-ups for the patients. WRA showed willingness for home-based mental health screenings by LHWs and expressed strong interest in group counselling sessions led by trusted LHWs.

LHWs, Lady Health Workers; WRA, Women of Reproductive Age.

### Pre-intervention phase

#### Development of counselling manuals

Manuals were designed by the research team of mPareshan,^23 24^ a study involving an app-based counselling intervention for flood-affected communities in Sindh. The content of this curriculum was adapted using the WHO Mental Health Gap Action Program-Intervention Guide V2.0 (mhGAP-IG 2.0).^25^ Three manuals were developed for this study: Improving Mental Health Awareness and Literacy: A Manual for Facility-Based Staff in Primary Care Settings, Improving Mental Health Awareness: A Manual for Frontline Community Health Workers Pakistan, and Responding to Climate Change – Community Awareness, Resilience-Building & First Response to Floods: A Trainer’s Manual for Frontline Workers to Build Resilience for Climate Change*.*

#### Hiring and training

The team for screening and delivering the intervention will include LHWs, LHS and BHU facility staff. The project will provide training to 20 LHWs in the intervention study arm to conduct household visits and administer screening tools. 12 female Senior Research Assistants (SRAs) along with four male SRAs will be recruited to assist.

The selected LHWs and LHS in the intervention clusters will receive training by the research team on screening using GAD-2 and PHQ-2, referral and provision of mental health awareness and resilience-building sessions. All BHU facility doctors will acquire training on assessment using GAD-7 and PHQ-9 of incoming referrals classified as screen-positive cases by LHWs, selection and administration of the most appropriate treatment strategies for mild or moderate cases (including counselling or psychoeducation for mild and moderate cases), and management and process of referral of severe cases for specialised care. Pre-training and post-training assessment of knowledge will be conducted for LHWs and LHS and communication and counselling skills for BHU or rural health clinic (RHC) facility staff.

#### Baseline assessment

A quantitative baseline will be conducted in the pre-intervention phase which will include a structured household survey to measure demographic information and screen for anxiety (GAD-7) and depression (PHQ-9). This component will focus on evaluating the feasibility of using LHWs for the purpose of screening and providing community interventions to WRA. By collecting baseline data, participants’ perceptions about mental health support and prior history regarding experiences of surviving floods will be assessed. This baseline survey will be carried out in both intervention and control arms. Informed consent will be acquired from all participants.

### Intervention phase

**Figure 3:**
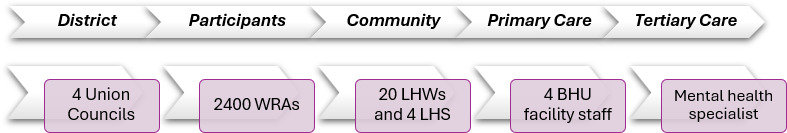
Tentative participants and data collectors during intervention phase. BHU, Basic Health Unit; LHS, Lady Health Supervisors; LHWs, Lady Health Workers; WRA, Women of Reproductive Age.

The intervention phase details are described below (figures3  4).

**Figure 4:**
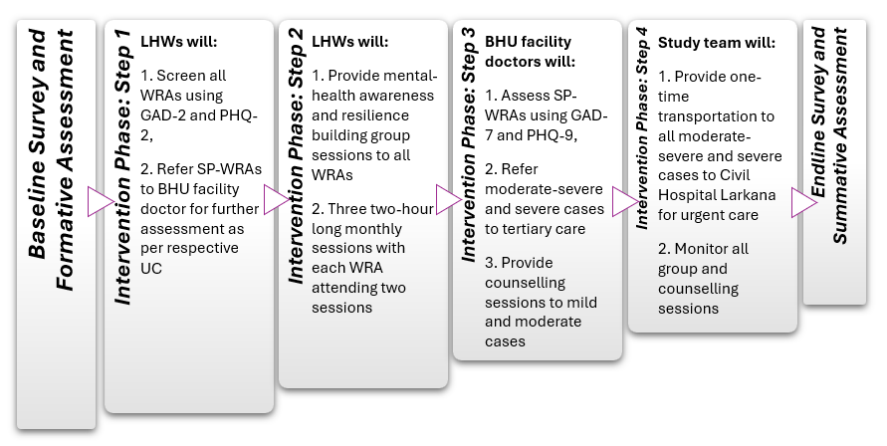
Data collection during intervention phase. BHU, Basic Health Unit; GAD-2, two-item Generalized Anxiety Disorder; GAD-7, 7-item Generalized Anxiety Disorder; LHWs, Lady Health Workers; PHQ-2, two-item Patient Health Questionnaire; PHQ-9, 9-item Patient Health Questionnaire; SP-WRAs, Screen-Positive Women of Reproductive Age; UC, Union Councils; WRA, women of reproductive age.

#### Intervention: Arm 1

The four UCs will receive community intervention provided by LHWs, LHS and BHU facility staff.

##### Implementation

The intervention involves several key components:

Step 1: Community-based mental health screening and referral by LHWs.

Trained LHWs will screen WRA using validated mental health tools, GAD-2 and PHQ-2.

- WRA scoring 3 or above on either tool will be classified as screen-positive for anxiety^16^ and/or depression.^17^
- A total of at least 2400 WRA will be screened across the intervention area, with each LHW responsible for approximately at least 120 WRA.
- SP-WRA will be issued standardised referral slips and directed to BHUs assigned to their respective UCs, where health facility-based doctors will conduct further screening using PHQ-9 and GAD-7 assessments.

Step 2: Awareness-raising and resilience-building group sessions by LHWs.

LHWs will conduct structured group sessions aimed at increasing mental health awareness, enhancing emotional resilience and fostering community support systems. These sessions will follow a standardised curriculum developed by the research team of mPareshan^23 24^ (Responding to Climate Change: Community Awareness, Resilience-Building and First Response to Floods, Appendix C).

- All WRA, regardless of their screening outcome, will participate in resilience-building group sessions to strengthen individual and community preparedness for future climate-related challenges.
- LHWs will provide three monthly sessions with each WRA attending two sessions, each lasting approximately 2 hours.
- Group sizes for each session will range from 20 to 25 WRA, ensuring interactive and supportive environments.
- LHS will monitor these sessions using a structured checklist to ensure fidelity to content and delivery standards.

Step 3: Facility-based counselling and referral pathways for screen-positive (SP) WRA.

WRA referred to BHU facilities will receive more comprehensive mental health assessments using the full PHQ-9 and GAD-7 tools. These assessments will be conducted by trained BHU doctors.

- WRA identified as experiencing mild to moderate symptoms of anxiety and/or mild to moderate symptoms of depression will be offered two individual counselling sessions, following the protocol outlined in Improving Mental Health Awareness and Literacy: A Manual for Facility-Based Staff in Primary Care Settings, Module 4. These sessions will be spaced bi-monthly, each lasting a minimum of 20 minutes, and scheduled according to patient and provider availability.
- WRA classified in the severe category of anxiety and/or moderate-severe to severe categories of depression will be urgently referred to mental health professionals at Civil Hospital Larkana for specialised care. The study team will provide one-time transportation support and necessary facilitation for these high-risk cases.

### Control: Arm 2

The control arm will include four Union Councils: Dhani Bux Bughio, Parya, Thalo and Sindhi Butera. These UCs will not receive any form of intervention other than standard routine care provided by LHWs.

### Compliance

Compliance will be maintained by record keeping through regular attendance data of mental health awareness and resilience-building group sessions conducted by LHWs, and referral information stored by health facility staff at BHUs. Geo-tagged pictures will be collected for all sessions to document attendance of WRA and LHWs. Findings will inform the feasibility, scalability and sustainability of integrating climate-adaptive mental health services within Pakistan’s primary healthcare system, particularly in vulnerable, disaster-prone communities.

### Quality assurance

LHS will monitor mental health and resilience-building group sessions conducted by LHWs using checklists to assess the quality of content relayed to WRA. BHU facility staff will be evaluated through consistent monitoring feedback forms collected from referred WRA after initial and follow-up visits. The study team will be involved in monitoring the group sessions and individual counselling sessions to maintain the quality of the intervention.

### Post-intervention phase

After the completion of resilience-building sessions in 4 months, an endline survey will be conducted across both intervention and control areas. The survey will include PHQ-9 and GAD-7 tools to assess changes in mental health status among WRA. In addition to the quantitative survey, qualitative data will be collected through FGDs and IDIs to gain insights into the barriers and facilitators of the intervention, as well as the broader community context.

The endline survey and summative study will serve to evaluate the impact of the intervention, focusing on shifts in mental health status among WRA, provide a comprehensive understanding of the intervention’s effectiveness and identify areas for improvement.

### Data collection

#### Monitoring, evaluation and cost assessment

Throughout the implementation period, detailed monitoring and cost-tracking will be conducted to assess the fidelity, reach and resource implications of the intervention (table 2). This includes data on:

**Table 2:** Study activities during implementation period

Study months and study activities	1	2	3	4	5	6	7	8	9	10	11	12
Formative research including data collection tools and application development (qualitative & quantitative surveys)	X	X	X									
Development of intervention components (manuals for LHWs, LHS and BHU facility staff)			X	X								
Recruitment & training (survey teams, BHU facility staff, LHWs, LHS)					X							
Baseline survey (household structured survey of WRA in intervention and control arms)					X							
Component A: LHW ultra-brief screening & referral (PHQ-2, GAD-2)						X						
Component B: LHW-led group mental health awareness & resilience-building sessions						X	X	X	X			
Component C: BHU assessment of referred SP-WRA (PHQ-9, GAD-7+case management)						X	X	X	X			
Endline PHQ-9 & GAD-7 administration (all WRA in intervention & control UCs)										X		
Data analysis											X	
Report writing & dissemination												X

BHU, Basic Health Unit; GAD-2, 2-item Generalized Anxiety Disorder; GAD-7, 7-item Generalized Anxiety Disorder; LHS, Lady Health Supervisors; LHWs, Lady Health Workers; PHQ-2, two-item Patient Health Questionnaire; PHQ-9, 9-item Patient Health Questionnaire; SP-WRA, screen-positive women of reproductive age; UCs, Union Councils; WRA, women of reproductive age.

- Screening coverage and referral completion.
- Group session attendance and quality.
- Counselling uptake and outcomes at BHU facilities.
- Overall resource utilisation and programmatic cost-efficiency.

Following the screening by LHWs and BHU facility staff, referrals will be tracked through the Data Management Unit (DMU) of Aga Khan University which will generate daily summary reports for quality check and, if required, send the reports to the field teams for rectification. All the data were encrypted, secured and fully anonymised.

#### Baseline and endline data collection

Participants will be assessed on baseline and endline levels involving data on sociodemographic factors, health-related information, household indicators, maternal health, trauma, impact of displacement and mental health, that is, depression and anxiety.

##### Demographic and socioeconomic indicators of households

Information regarding socioeconomic status, gender, level of education, marital status, number of children, occupation of the household head, water, sanitation and hygiene (WASH), household infrastructure material, and financial and economic assets will be recorded.

##### Maternal health, diseases and morbidity

Types of diseases, duration, maternal health and pregnancy-related information will be collected.

##### Displacement and trauma

Information will be collected regarding fear and trauma experienced by WRA after surviving flooding involving loss of loved ones, financial and economic assets, houses, and agricultural livelihoods.

##### Mental health screening (GAD-7 and PHQ-9)

Screening tools that will be used for assessing symptoms of depression and anxiety are the GAD-2,^16^ PHQ-2,^17^ GAD-7^18^ and PHQ-9.^19^ All tools are reliable and valid for screening purposes^16^,^19^ and were translated into Sindhi language by a minimum of two native Sindhi-speaking researchers.

### Patient and public involvement

Stakeholders from the Sindh Lady Workers Programme and the District Health Department, and community members were involved in the design of the trial, as mentioned in the formative phase. Their perceptions and opinions regarding mental health screening and referral, and resilience-building sessions were used in the formation of the intervention, which may subsequently benefit the community.

### Data analysis

#### Quantitative component

The data collected and stored on the server will be exported to Stata version 18 for analysis. Data will be cleaned, and errors will be rectified under the guidance of the study investigators. Descriptive statistics will be presented: mean and SD, or median with IQRs for continuous variables, depending on the data distribution, and frequency distributions for categorical variables. Feasibility, acceptability and appropriateness of the intervention were assessed through proportions with 95% CIs. For mental health outcomes, difference-in-difference (DID) analyses will be conducted through logistic regression analysis, assuming parallel trends i.e., intervention and control arms had parallel trends in the outcomes before the intervention was introduced. The model incorporates an interaction term for time and intervention effect. The coefficient of the interaction term serves as DID estimate. Further, to address potential confounding, the DID will be adjusted for imbalanced factors at baseline. In addition, the model will also include covariates that are known to affect the outcome such as age, education, marital status, parity, socioeconomic status and trauma. For missing data on variables, the plan will be not to impute but treat this as missing and perform complete case analysis. All analysis will be adjusted to account for the cluster randomised design.

#### Qualitative component

For the qualitative phase, thematic content analysis will be employed to systematically analyse data from FGDs and IDIs, identifying key themes and patterns that provide deeper insights into the intervention’s effectiveness, barriers and facilitators.

## Ethics and dissemination

### Ethical considerations

The study acquired ethical approval from the Ethical Review Committee at Aga Khan University (2024-10475-30776) and the National Bioethics Committee (4-87/NBC-1158/23/481) in Islamabad. The Ethics Review Committee (ERC) and health department will be informed of any modifications made to the protocol of the trial. Approval was obtained from relevant provincial authorities involved. The trial will adhere to the ethical principles of autonomy, anonymity, confidentiality, equity and respect. All eligible participants will be provided with informed consent, details regarding the purpose and procedure of the study, and the right to withdraw at any time. Collaboration will be maintained with the Sindh Lady Workers Programme and the District Health Department. Community engagement events will be conducted regularly to address concerns and encourage participation, with serious issues resolved through the community Jirga system. Severe cases of anxiety and depression will be referred to tertiary care with one-time transport provided by the study team. Coordination with tertiary care staff is in place to ensure seamless and prioritised care for referred individuals. A distress protocol will be established to support any participant or data collector experiencing any form of psychological distress due to the content of the study.

### Data management and dissemination

Data will be anonymised and securely stored, with protection protocols in place. The de-identified data will be shared at reasonable request and approval from the institute and the funding authority. The methods and outcomes of the study will be published in peer-reviewed journals. Dissemination of the results of the trial will occur after its completion to stakeholders, participants and the public.

## Discussion

Climate change-related disasters have become an alarming threat to the physiological and psychological health of individuals across the world. Worsened by climate change, floods have made people more susceptible to a variety of illnesses and financial crises.^26 27^ Additionally, low-middle income countries are already at a high risk of mental health issues due to the scarcity of resources and necessities including provision of mental health services.^28^ Coupled with the catastrophic floods of 2022 in Pakistan’s context, there have been a myriad of common mental health disorders significantly increasing in these affected areas.^27^

A protective factor which can combat the negative impact of floods and natural disasters on the mental health of those affected is social support.^29 30^ A study in Punjab’s flood-affected areas noted that higher social support was associated with lower levels of anxiety, stress and depression.^26^ Consequently, in this trial, community interventions will be incorporated to build climate change resilience and spread awareness regarding mental health among the flood-affected areas of Sindh. The focus of the study remains on women of reproductive age as they seem to be the most susceptible to the negative impact of floods.^27^ Even as per the findings of the formative phase of this trial, women suffered due to the absence of security, privacy and basic healthcare required, especially during pregnancy.

This contextually grounded, evidence-based intervention addresses the urgent and growing mental health needs of WRA in Pakistan’s climate-vulnerable regions. By embedding mental health services within the trusted LHW network and aligning with existing primary care structures, this initiative offers a sustainable and scalable solution if proven to be effective. It aims not only to alleviate psychological distress but also to foster resilience, restore agency and promote well-being in communities facing intersecting challenges of environmental stress and socioeconomic adversity.

## Supplementary material

10.1136/bmjopen-2025-104759 online supplemental file 1

10.1136/bmjopen-2025-104759 online supplemental file 2

10.1136/bmjopen-2025-104759 online supplemental file 3

10.1136/bmjopen-2025-104759 online supplemental file 4

10.1136/bmjopen-2025-104759 online supplemental file 5

10.1136/bmjopen-2025-104759 online supplemental file 6

10.1136/bmjopen-2025-104759 online supplemental file 7

10.1136/bmjopen-2025-104759 online supplemental file 8

10.1136/bmjopen-2025-104759 online supplemental file 9

10.1136/bmjopen-2025-104759 online supplemental file 10

10.1136/bmjopen-2025-104759 online supplemental file 11

10.1136/bmjopen-2025-104759 online supplemental file 12
