# Supplementary material for: Community-based mental health screening & referral for flood-affected women in rural Pakistan: an intervention feasibility study protocol
Source: BMJ Open. 2025 Oct 23;15(10):e104759. doi: 10.1136/bmjopen-2025-104759 (PMC12551463; doi:10.1136/bmjopen-2025-104759)
Supplement: online supplemental file 1 [file bmjopen-15-10-s001.docx]

**Community-Based Mental Health Screening & Referral for Flood-Affected Women in Dadu: A Feasibility Study**

**Qualitative Component**

**FGD Guide for Pre-Intervention for Community Participants (WRAs)**

| **Guidelines for Formative phase Focus Group Discussion (FGD) with Community Participant (CP)** **i.e. Women of Reproductive Age (WRA)**  **Consent**: Written informed consent will be signed by each participant before commencing the FGD.  **Duration**: 45 minutes will be allocated, or it can be extended until the point of saturation  **Mode of recording**: Tape recorder will be used for recording. In addition, written notes will also be taken.  **Place for interview**: LHW Program Office OR participant’s home/community venue if coming to LHW-P office is not possible.  **Transcription**: Following the completion of each discussion, tape verbatim will be transcribed, noting pauses, changes in tone, laughter, and moderator’s questions, comments, and affirmative “noises.” In addition, length of FGD and amount of time required to transcribe will also be noted at the end of transcript, so that other FGDs can be modified or implemented accordingly.  FGD will be conducted by a team of two. One person will moderate the session, and the other will record the responses, both in writing and by audio recorder.  **General instructions**   - **Welcome the participants.** - **Overview of the topic:** The overall aim of the study is to demonstrate that in already vulnerable populations further affected and displaced by climate change-related crises such as mass flooding, mental health screening and referral can be successfully implemented by community health workers, along with community-level education/awareness sessions and other activities designed to build community, household, and individual-level resilience to the effects of climate change, including the mental health effects. - **Purpose of the FGD:** The purpose of this FGD is to explore community participant’s views regarding burden of mental illness in their community, capacity of LHWs to deliver home-based mental health screening, referral, and awareness/resilience-building services. Additionally, the FGD will probe perceptions about the feasibility of uptake of these services by LHW-P and women of reproductive age (WRA) in this flood-affected community. This FGD will also take community participant’s opinions regarding how LHSs can provide effective supportive supervision to LHWs during the intervention roll out.   **Ground rules of FGD**   - Please talk in a loud voice. - Kindly feel free not to respond to questions that you cannot relate to and feel uncomfortable answering. - Please ask questions/clarification as they come up. |
| --- |

FGD session No: ________________

**Session attendance information sheet** (To be filled by participants)

| **S. No** | **Name of CP** | **Language(s) Spoken** | **Age (years)** | **Occupation** | **Work experience (in years, if any)** | **Contact details** | **Education level (matric, intermediate, university degree, post-graduate qualification)** |  |
| --- | --- | --- | --- | --- | --- | --- | --- | --- |
| 1. |  |  |  |  |  |  |  |  |
| 2. |  |  |  |  |  |  |  |  |
| 3. |  |  |  |  |  |  |  |  |
| 4. |  |  |  |  |  |  |  |  |
| 5. |  |  |  |  |  |  |  |  |
| 6. |  |  |  |  |  |  |  |  |
| (To be filled by moderator)  **Date of FGD: __/__/____ Duration of FGD: ______**  **UC Name:**  **Village Name:**  **Place of FGD Begin - __: __**    **Name of moderator: End - __: __**    **Name of note taker:** | | | | | | | | |

| **S. No.** | **Lead** | **Comments** |
| --- | --- | --- |
| **Participant’s perception about mental health and mental health service provision** | | |
|  | What do you understand by ‘mental health’?  Probes:   - What sources do you get information on mental health from? - What is ‘good’ mental health vs ‘poor’? - How do you judge someone’s mental health? |  |
|  | How is the overall mental health of the community?  Probes:   - How common are mental health issues in the community? - How are WRA (women of reproductive age) affected? |  |
|  | Are you aware of any available mental health services within your community?  Probes:   - What are the available resources? - How accessible are these resources? - How is your experience like using these services? - Do you face any stigma and/or discrimination while using such services? |  |
| **Perception about impact of climate change on mental health, and availability of disaster management services** | | |
|  | What comes to your mind when you hear the term ‘climate change’?  Probes:   - How do you think climate change happens/what causes climate change? - Has climate change impacted any part of your life? If yes, please describe how. If not, please explain why not. |  |
|  | Is there any link between climate change and the floods you experienced in 2022?  Probes:   - Were there any protective/preventative measures in place by the community? - How did the community handle displacement because of the floods? - Please describe your lived experience of the floods. What difficulties did you face? |  |
|  | How did the community react to the floods?  Probes:   - Difficulties faced by the community during the floods - Difficulties faced by WRAs |  |
|  | How did the disaster management services respond to the floods in 2022?  Probes:   - What resources were available to the displaced? - How prepared/ready were the disaster management services in responding to the floods? |  |
|  | What impact did the floods of 2022 have on your mental health and the mental health of the community?  Probes:   - What were you most worried about when the floods hit your community? - Did you feel dejected or dispirited during the entire experience? - What other emotions/feelings did you experience during the floods and in their aftermath? |  |
| **Acceptability and appropriateness of LHWs for providing mental health screening & referral** | | |
|  | Please tell us how you feel about the LHW program.  Probes:   - How is the quality of care delivered like? - What is your level of satisfaction with the program services? |  |
|  | What is the level of comfort within the community in talking to LHW about their mental health status? |  |
|  | Do you think that LHWs should include home-based mental health screening during visits?  Probes:   - Do you think that they can effectively and competently complete a rapid assessment? Why/why not? - Do you think the community would participate in this screening? Why/why not? - Competency in noting symptoms/data collection |  |
|  | If an LHW refers you to a BHU/RHC, how likely are you going to visit?  Probes:   - Will you visit the referral facility if LHW recommends? - What factors determine your decision to visit? - What is your experience visiting a BHU/RHC in the past? - Do you think that the BHU/RHC staff have the capability to provide dedicated mental health services? |  |
| **Ability of LHWs to deliver group mental health awareness and resilience-building sessions in community** | | |
|  | How likely is it that you would attend sessions conducted by LHWs on mental health awareness and resilience building against climate change?  Probes:   - Do you think that LHWs are capable to deliver such information? - What do you hope to get out of such sessions? - Effectiveness of sessions - Will it change your resilience to climate change? - What benefit do you foresee from such group sessions? |  |

We have reached the end of our interview. Thank you for your participation.
