# Supplementary material for: Community-based mental health screening & referral for flood-affected women in rural Pakistan: an intervention feasibility study protocol
Source: BMJ Open. 2025 Oct 23;15(10):e104759. doi: 10.1136/bmjopen-2025-104759 (PMC12551463; doi:10.1136/bmjopen-2025-104759)
Supplement: online supplemental file 2 [file bmjopen-15-10-s002.docx]

**Community-Based Mental Health Screening & Referral for Flood-Affected Women in Dadu: A Feasibility Study**

**Qualitative Component**

**FGD Guide for Post-Intervention for Community Participants (WRAs)**

| **Guidelines for post-intervention Focus Group Discussion (FGD) with Community Participants i.e. Women of Reproductive Age (WRA)** who have received intervention through community-based mental health screening and referral by LHWs.  **Consent**: Written consent form will be signed/thumb impression taken for the participant before commencing the FGD.  **Duration**: 45 minutes will be allocated, or it can be extended until the point of saturation.  **Mode of recording**: A tape recorder will be used for recording the interview. In addition, written notes will also be taken during the interview.  **Place for interview**: LHW Program Office OR participant’s home/community venue if coming to LHW-P office is not possible.  **Transcription**: Following the completion of each discussion, tape verbatim will be transcribed, noting pauses, changes in tone, laughter, and moderator’s questions, comments, and affirmative “noises.” In addition, length of FGD and amount of time required to transcribe will also be noted at the end of transcript, so that other FGDs can be modified or implemented accordingly.  FGD will be conducted by a team of two researchers. One person will moderate the session, and the other will record the responses, both in writing and by audio recorder.  **General instructions**   - **Welcome the participant** - **Overview of the topic:** The overall aim of the study is to demonstrate that in already vulnerable populations further affected and displaced by climate change-related crises such as mass flooding, mental health screening and referral can be successfully implemented by community health workers, along with community-level education/awareness sessions and other activities designed to build community, household, and individual-level resilience to the effects of climate change, including the mental health effects. - **Purpose of the FGD:** The purpose of FGD is to explore community participants’ views regarding intervention uptake in the community, and the barriers and facilitators they faced during implementation roll out. These community participants have took part in the mental health screening and referral intervention, as well as being referred to referral facility (BHU/RHC) staff for further mental health screening and counselling.   **Ground rules of FGD**   - Please talk in a loud voice. - Kindly feel free not to respond to questions that you cannot relate to and feel uncomfortable answering. - Please ask questions/clarification as they come up. - Kindly respect each other’s opinion |
| --- |

FGD session No: ________________

**Session attendance information sheet** (To be filled by participants)

| **S.No.** | **Name of CP** | **Age (yrs)** | **Work experience (if any)** | **Contact details** | **Education level (matric, intermediate, university degree, post-graduate qualification)** | **Completed the intervention?** |
| --- | --- | --- | --- | --- | --- | --- |
| 1. |  |  |  |  |  | Yes/No |
| 2. |  |  |  |  |  | Yes/No |
| 3. |  |  |  |  |  | Yes/No |
| 4. |  |  |  |  |  | Yes/No |
| 5. |  |  |  |  |  | Yes/No |
| 6. |  |  |  |  |  | Yes/No |
| (To be filled by moderator)  **Date of FGD: __/__/____ Duration of FGD: ______**  **UC Name:**  **Village Name:**  **Place of FGD Begin - __: __**    **Name of moderator: End - __: __**    **Name of note taker:** | | | | | | |

| **S. No.** | **Lead** | **Comments** |
| --- | --- | --- |
| **Intervention Uptake** | | |
|  | What do you understand by ‘mental health’?  Probes:   - What sources do you get information on mental health from? - What is ‘good’ mental health vs ‘poor’?   How do you judge someone’s mental health |  |
|  | How useful were the mental health screening and referral in community-based settings to improve mental healthcare service delivery?  Probes:   - Can LHWs successfully screen for mental health symptoms? - What are your reasons for agreeing/disagreeing? - How useful is this service? |  |
|  | What was your interaction with LHWs during screening and referral like?  Probes:   - Was the mode of delivery likeable? - Were you comfortable getting screened by LHWs? - Was the referral pathway smooth? - Was the time between screening and referral, okay? - Any recommendations for the future? |  |
|  | How was your experience of group mental health awareness and resilience building sessions?  Probes:   - How engaging did you find the content? - Did it help in raising your mental health awareness? - Did it help in raising your resilience against climate change (especially dealing with flood-related disasters) - Is raising your awareness of mental health helpful in dealing with distress caused by flood-related issues? - Was the group session enough? - Would you recommend other WRA in your community to attend the session? |  |
|  | What are your views regarding LHWs delivering group mental health awareness and resilience-building sessions?  Probes:   - Did you like this mode of delivery? - Were the LHWs capable of delivering the group session? - Were they supervised by LHSs? - Any recommendations? |  |
|  | How was your experience of attending referral facility (BHU/RHC) for further mental health screening and counselling?  Probes:   - Was it easy to go to referral facility? - Did you find it useful to be referred to a facility after group session by LHWs? - Reasons for agreeing/disagreeing - Was space available for your counselling? Was it private enough? |  |
|  | What are your views regarding referral facility staff conducting mental health screening and counselling?  Probes:   - Did you find the referral facility staff’s screening more useful than LHWs? - Reasons for agreeing/disagreeing - Were the staff available when you came to the facility? - Were resources available to you (time, space, other facilities?) - Was your data managed/record kept successfully? - Did you find counselling at BHU/RHC level useful? - Did it help in improving your mental health symptoms - What part was the most useful? - Would you recommend other WRA in your community (who have mental health problems) to come to the referral facility? - Any recommendations? |  |
|  | Please tell us your perspective on benefit of intervention for mental health service provision and raising community resilience to climate change.  Probes:   - Will this help mental health conditions in your district in the future? - Will this help make the community more resilient to climate change? - Do you think giving this intervention to WRAs is most useful (as they are affected the most) or it can be expanded to general adult population? |  |
| **Barriers to implementation** | | |
|  | Please outline the factors that created barriers to implementation  Probes:   - Problems faced during screening and referral - Problems faced during group session - Problems faced in going to referral facility and receiving counselling - Any reservations about intervention? |  |
|  | Suggestions for improvement and sustainability |  |
| **Facilitators to Implementation** | | |
|  | What were the facilitators during the intervention roll out?  Probes:   - Role of LHWs in delivering screening, referral and then group sessions on awareness raising. - Role of community in taking up intervention - What were the best features of the intervention? - What factors facilitated successful delivery? |  |

We have reached the end of our interview. Thank you for your participation. Do you have any additional suggestions for the LHW-P and BHU/RHC staff in continuing this intervention?
