# Supplementary material for: Community-based mental health screening & referral for flood-affected women in rural Pakistan: an intervention feasibility study protocol
Source: BMJ Open. 2025 Oct 23;15(10):e104759. doi: 10.1136/bmjopen-2025-104759 (PMC12551463; doi:10.1136/bmjopen-2025-104759)
Supplement: online supplemental file 4 [file bmjopen-15-10-s004.docx]

**Community-Based Mental Health Screening & Referral for Flood-Affected Women in Dadu: A Feasibility Study**

**Qualitative Component**

**FGD Guide for Post-Intervention for LHWs**

| **Guidelines for post-intervention Focus Group Discussion (FGD) with Lady Health Workers (LHWs)** who took part in the intervention delivery  **Consent**: Written informed consent will be signed by each participant before commencing the FGD.  **Duration**: Nearly 45 minutes will be allocated, or it can be extended until the point of saturation.  **Mode of recording**: Tape recorder will be used for recording. In addition, written notes will also be taken.  **Place for FGD**: LHW-P office where participants feel comfortable, and their privacy will be ensured.  **Transcription**: Following the completion of each discussion, tape verbatim will be transcribed, noting pauses, changes in tone, laughter, and moderator’s questions, comments, and affirmative “noises.” In addition, length of FGD and amount of time required to transcribe will also be noted at the end of transcript, so that other FGDs can be modified or implemented accordingly. FGD will be conducted by a team of two researchers. One person will moderate the session, and the other will record the responses, both in writing and by audio recorder.  **General instructions**   - **Welcome the participants** - **Overview of the topic:** The overall aim of the study is to demonstrate that in already vulnerable populations further affected and displaced by climate change-related crises such as mass flooding, mental health screening and referral can be successfully implemented by community health workers, along with community-level education/awareness sessions and other activities designed to build community, household, and individual-level resilience to the effects of climate change, including the mental health effects. - **Purpose of the FGD:** The purpose of FGD is to explore LHWs’ experiences regarding uptake of the intervention and the barriers and facilitators faced during implementation roll out.   **Ground rules of FGD**   - Please talk in a loud voice. - Kindly feel free not to respond to questions that you cannot relate to and feel uncomfortable answering. - Please ask questions/clarification as they come up. - Kindly respect each other’s opinion |
| --- |

FGD session No: ________________

**Session attendance information sheet** (To be filled by participants)

| **S. No.** | **Name of LHS** | **Age (yrs)** | **Catchment area/taluka** | **Work experience (yrs)** | **Contact details.** | **Education level (matric, intermediate, university degree, post-graduate qualification)** |
| --- | --- | --- | --- | --- | --- | --- |
|  |  |  |  |  |  |  |
|  |  |  |  |  |  |  |
|  |  |  |  |  |  |  |
|  |  |  |  |  |  |  |
|  |  |  |  |  |  |  |
|  |  |  |  |  |  |  |
|  | (To be filled by moderator)  **Date of FGD: __/__/____ Duration of FGD: ________**  **UC Name:**  **Village Name:**  **Place of FGD Begin - __: __**  **Name of moderator: End - __: __**  **Name of note taker:** | | | | | |

| **S. No.** | **Lead** | **COMMENTS** |
| --- | --- | --- |
| **Intervention uptake** | | |
|  | What do you understand by ‘mental health’?  Probes:   - How do you judge someone’s mental health?   What is ‘good’ mental health vs ‘poor’? |  |
|  | How did the WRAs receive the mental health screening and referral facilities?  Probes:   - How was your experience of conducting screening and referral during routine visits? - How do you feel about supportive supervision by LHSs during screening and referral? - Was there an ease of communication between LHW and LHS for referral cases/screening scores? - Were the WRAs satisfied or unsatisfied? Why? |  |
|  | How did the community receive to provision of mental health screening and referral facilities provided to them at home?  Probes:   - Was the community satisfied or unsatisfied? Why? - What did the participants like the most? And what did they dislike the most? - How much time were participants willing to give? - time availability of participants |  |
|  | What role did the supervision of LHS’ play in the intervention roll out?  Probes:   - What impact did the supervision have on the intervention? How did the LHS facilitate the intervention roll out? |  |
|  | How did you feel about the screening and referrals services?  Probes:   - Confidence in using screening tools - Time and willingness during routine visits - Participant’s responsiveness to referral - Difficulties in asking questions related to mental health |  |
|  | How did you feel about the effectiveness of using brief and rapid screening tools and referrals to BHUs/RHCs for specialist care?  Probes:   - How effective were the screening tools? - What was the uptake rate for referrals? |  |
|  | Were the group mental health awareness and resilience-building sessions useful in any way? Please elaborate on why.  Probes:   - Was there an effective utilization of LHWs’ time? - Was there significant community engagement? - usefulness of sessions in reducing mental health symptoms, - usefulness of session in increasing community resilience to climate change (floods) - impact of sessions in increasing awareness about dealing with natural disasters |  |
|  | How useful was the referral service provided by the LHWs?  Probes:   - Did referral to specialized care facility increase because of LHWs’ screening? - Were referral cases catered to sufficiently at referral facility? - What factors contributed to acceptance/rejection of being referred in community WRAs? |  |
| **Barriers to implementation** | | |
|  | What were the barriers that LHW’s experienced during the intervention roll out?  Probes:   - Problem between LHW/LHS coordination - Problems engaging with community - Problems faced between LHW and referral cases - Problems faced during group session delivery - Any other barriers? |  |
|  | Suggestions for improvement and sustainability |  |
| **Facilitators to Implementation** | | |
|  | What were the facilitators that LHWs experienced during intervention roll out?  Probes:   - Role of supportive supervision - Role of community - Role of referral facility staff - Role of study team - Any other facilitators? |  |

We have reached the end of our interview. Do you have any additional suggestions for LHWs conducting mental health screening and referral in flood-affected areas?
