# Supplementary material for: Community-based mental health screening & referral for flood-affected women in rural Pakistan: an intervention feasibility study protocol
Source: BMJ Open. 2025 Oct 23;15(10):e104759. doi: 10.1136/bmjopen-2025-104759 (PMC12551463; doi:10.1136/bmjopen-2025-104759)
Supplement: online supplemental file 5 [file bmjopen-15-10-s005.docx]

**Community-Based Mental Health Screening & Referral for Flood-Affected Women in Dadu: A Feasibility Study**

**Qualitative Component**

**Interview Guide for Pre-Intervention for Disaster Management Representatives**

| **Guidelines for post-intervention Key Informant Interviews (KII) with Disaster Management representatives.**  One semi-structured questionnaire will be used for each participant who has consented.  **Consent**: Written consent form will be signed by each participant before commencing each key informant interview.  **Duration**: 30 minutes will be allocated, or it can be extended until the point of saturation.  **Mode of recording**: A tape recorder will be used for recording each key informant interview. In addition, written notes will also be taken during the interview  **Place for interview**: Office of the participant/AKU/online over zoom whatever is feasible.  **Transcription**: Following the interview, tape verbatim will be transcribed, noting pauses, changes in tone, laughter, comments, and affirmative “noises.” In addition, the length of the interview and amount of time required to transcribe will also be noted at the end of the transcript, so that other key informant interviews can be modified or implemented accordingly.  The interview will be conducted by a team of two researchers. One person will ask the questions, and the other will record the responses, both in writing and with an audio recorder.  **General instructions**   - **Welcome the participant.** - **Overview of the topic:** The overall aim of the study is to demonstrate that in already vulnerable populations further affected and displaced by climate change-related crises such as mass flooding, mental health screening and referral can be successfully implemented by community health workers, along with community-level education/awareness sessions and other activities designed to build community, household, and individual-level resilience to the effects of climate change, including the mental health effects. - **Purpose of the KII:** The purpose of KII is to explore disaster management representative’s views regarding uptake of intervention and barriers and facilitators to implementation roll out.   **Ground rules of KII**   - Please talk in a loud voice. - Kindly feel free not to respond to questions that you cannot relate to and feel uncomfortable answering. - Please ask questions/clarification as they come up. |
| --- |

KII session No: ________________

**PARTICIPANT’S INFORMATION: to be filled by interviewer**

| Name of disaster management official |  |
| --- | --- |
| Gender |  |
| Age |  |
| Designation |  |
| Place of work or institution |  |
| Work experience |  |
| Qualification |  |
| Contact details |  |

To be filled in by the interviewer:

| Name of Interviewer |  |
| --- | --- |
| Name of note taker |  |
| Duration of interview | Begin End |
| Date of Interview | DD / MM/ YY |

| **S. No.** | **Lead** | **Comments** |
| --- | --- | --- |
| **Uptake of intervention** | | |
|  | What are your views regarding LHWs doing mental health screening and referral for women of reproductive age (WRA) in community?  Probes:   - Were the LHWs capable of performing the screenings and referrals? - Did this screening and referral intervention help flood-affected WRAs? |  |
|  | What are your views regarding LHWs delivering group mental health and resilience building awareness session in community?  Probes:   - Did this session help raise community resilience to climate change? - Can disaster management services be supplemented by group sessions like these delivered at community doorsteps? - Having group sessions like these is acceptable in the community? |  |
|  | What are your views regarding benefits of intervention for mental health service provision and community resilience to climate change  Probes:   - Do disaster management authorities want to be involved in such group sessions in the future? - Is raising mental health awareness beneficial for climate change resilience? - How successful do you think this strategy was? |  |
| **Barriers to implementation roll out** | | |
|  | What are your views on barriers to implementation roll out? |  |
|  | Do you have any suggestions for improvement and sustainability?  Probes:   - Can disaster management authorities provide more support for building community resilience to climate change? - Do they have resources? - Resiliency against floods – was this the best district to pilot this strategy? |  |
| **Facilitators to implementation roll out** | | |
|  | What are your views on facilitators for implementation roll out  Probes:   - What was the role of LHW’s acceptability in community to deliver such intervention? - Short-term intervention to impart long-term resilience against climate events/natural disasters (is this doable?) |  |
|  | How feasible was this strategy?  Probes:   - Can this strategy work in the future? - Should more modules on climate change resilience be added to group awareness sessions (for e.g. dealing with extreme heat etc)? |  |

We have reached the end of our interview. Thank you for your participation. Do you have any further feedback?
