# Supplementary material for: Community-based mental health screening & referral for flood-affected women in rural Pakistan: an intervention feasibility study protocol
Source: BMJ Open. 2025 Oct 23;15(10):e104759. doi: 10.1136/bmjopen-2025-104759 (PMC12551463; doi:10.1136/bmjopen-2025-104759)
Supplement: online supplemental file 6 [file bmjopen-15-10-s006.docx]

**Community-Based Mental Health Screening & Referral for Flood-Affected Women in Dadu: A Feasibility Study**

**Qualitative Component**

**FGD Guide for Pre-Intervention for LHWs**

| **Guidelines for Formative phase Focus Group Discussion (FGD) with Lady Health Workers (LHWs)**  **Consent**: Written informed consent will be signed by each participant before commencing the FGD.  **Duration**: 45 minutes will be allocated, or it can be extended until the point of saturation  **Mode of recording**: Tape recorder will be used for recording. In addition, written notes will also be taken.  **Place for FGD**: LHW-P office/any other designated official space where participants feel comfortable, and their privacy can be ensured.  **Transcription**: Following the completion of each discussion, tape verbatim will be transcribed, noting pauses, changes in tone, laughter, and moderator’s questions, comments, and affirmative “noises.” In addition, length of FGD and amount of time required to transcribe will also be noted at the end of transcript, so that other FGDs can be modified or implemented accordingly. FGD will be conducted by a team of two researchers. One person will moderate the session, and the other will record the responses, both in writing and by audio recorder.  **General instructions**   - **Welcome the participants.** - **Overview of the topic:** The overall aim of the study is to demonstrate that in already vulnerable populations further affected and displaced by climate change-related crises such as mass flooding, mental health screening and referral can be successfully implemented by community health workers, along with community-level education/awareness sessions and other activities designed to build community, household, and individual-level resilience to the effects of climate change, including the mental health effects. - **Purpose of the FGD:** The purpose of this FGD is to explore LHWs’ views regarding the burden of mental illness in their community, capacity of LHWs to deliver home-based mental health screening, referral, and awareness/resilience-building services. Additionally, the FGD will probe LHWs’ perceptions about the feasibility of uptake of these services by LHW-P and women of reproductive age (WRA) in this flood-affected community. This FGD will also take LHWs’ opinion regarding how LHSs can provide effective supportive supervision to LHWs during the intervention roll out.   **Ground rules of FGD**   - Please talk in a loud voice. - Kindly feel free not to respond to questions that you cannot relate to and feel uncomfortable answering. - Please ask questions/clarification as they come up. |
| --- |

| **S. No.** | **Lead** | **COMMENTS** |
| --- | --- | --- |
| **Mental health disease burden, availability of mental health and disaster management services in community** | | |
|  | What do you understand by ‘mental health’?  Probes:   - What sources do you get your information on mental health from? How do you judge someone’s mental health? - How do they think mental health challenges manifest themselves in people? - What is ‘good’ mental health vs ‘poor’? |  |
|  | How is the overall mental health of the community?  Probes:   - How do people in the community describe mental health? - How common are mental health issues in the community? - How do community members and elders recognize mental health problems? How do they react to those suffering from mental health challenges? Are WRA (women of reproductive age) affected and how? |  |
|  | Can you please outline any mental health services within your community?  Probes:   - What are the available resources? - How accessible are these resources? - How is the experience of others like using these services? - Do they face any stigma and/or discrimination while using such services? Do you think the existing resources are sufficient to deal with the problem? |  |
|  | How likely will the community make use of home-based mental health services delivered by LHWs?  Probes: Community factors leading to acceptance or rejection   - What barriers exist to the uptake of such services? - What facilitators exist to the uptake of such services? |  |
|  | How did the community react to the floods?  Probes:   - What difficulties were faced during the floods? - What difficulties did WRAs face? - Were there any protective/preventative measures in place by the community? - How did the community handle displacement because of the floods? |  |
|  | What impact did the floods of 2022 have on your mental health and the mental health of the community?  Probes:   - What were you and others in your community most worried about when the floods hit your community? - What emotions/feelings did you experience during the floods and in their aftermath? |  |
|  | How did the disaster management services respond to the floods in 2022?  Probes:   - What resources were available to the displaced? - How prepared/ready were the disaster management services in responding to the floods? |  |
| **Acceptability and appropriateness of LHWs for mental health screening & referral** | | |
|  | What is your relationship with the community like?  Probes:   - Relationship with community WRAs, - WRA problems and lived experiences. - Did this relationship change in any way during or after the floods? - Did LHWs play any role during the floods? |  |
|  | Do you think you can effectively deliver mental health services during home visits?  Probes:   - What capability do you think is required for effective delivery? - What are the factors for willingness or reluctance to take on mental health service delivery? |  |
|  | Do you think you can effectively screen for mental health problems during home visits?  Probes:   - Competency - What capability do you think is required for effective delivery? - How has your experience with other screening tools been? |  |
|  | Do you think you can easily make referrals to BHUs/RHCs for mental health problems in the community?  Probes:   - Confidence in making referrals,   Acceptance in community for referring to BHU/RHCs |  |
|  | Do you think this intervention would be accepted by LHWs and the community alike?  Probes:   - What barriers could this intervention face? - What existing strengths can this intervention build upon? |  |
| **Ability of LHWs to deliver group mental health awareness and resilience-building sessions** | | |
|  | What comes to your mind when you hear the term ‘climate change’?  Probes:   - How do you think climate change happens/what causes climate change? - Has climate change impacted any part of your life? If yes, please describe how. If not, please explain why not. |  |
|  | Is there any link between climate change and the floods you experienced in 2022?  Probes:   - Available resources to manage flood-affected issues, - Readiness to deal with floods/natural disasters - Please describe your lived experience of the floods. What difficulties did you face? - Effect on routine LHW service delivery |  |
|  | Do you think there is any link between climate change and mental health?  Probes:   - Mental distresses caused by the floods. - Lived experiences during floods. - How can the community build mental and physical resiliency in the face of climate change? - How can the community build mental and physical resiliency in the face of natural disasters? |  |
|  | Do you think you can effectively be trained on and deliver community-focused sessions on mental health awareness and resilience-building to WRAs?  Probes:   - What existing factors would lead to willingness or reluctance to receive training? - How confident do you think LHWs would be to deliver such sessions? - Are there any barriers or facilitating factors to effectively deliver such sessions? |  |
|  | How likely is it that such sessions will be readily accepted and supported by the community?  Probes:   - At community level- will WRAs accept such services - At LHW level: will LHWs accept this mode of service delivery. - At LHW level: will they accept LHSs’ supervision during group awareness raising sessions - Potential hurdles during delivery of group sessions (venue, getting WRAs to group session, attendance, level of understanding in community) |  |

We have reached the end of our interview. Thank you for your participation.
