# Supplementary material for: Community-based mental health screening & referral for flood-affected women in rural Pakistan: an intervention feasibility study protocol
Source: BMJ Open. 2025 Oct 23;15(10):e104759. doi: 10.1136/bmjopen-2025-104759 (PMC12551463; doi:10.1136/bmjopen-2025-104759)
Supplement: online supplemental file 7 [file bmjopen-15-10-s007.docx]

**Community-Based Mental Health Screening & Referral for Flood-Affected Women in Dadu: A Feasibility Study**

**Qualitative Component**

**Interview Guide for Pre-Intervention for Disaster Management Representatives**

| **Guidelines for Formative Phase Key Informant Interviews with Disaster Management (DM) representatives**  One semi-structured questionnaire will be used for each participant who has consented.  **Consent**: Written consent form will be signed by the participant before commencing each key informant interview.    **Duration**: 30 minutes will be allocated, or it can be extended until the point of saturation.  **Mode of recording**: A tape recorder will be used for recording the key informant interview. In addition, written notes will also be taken during the interview.  **Place for interview**: Office of the participant/AKU/online over zoom whatever is feasible.  **Transcription**: Following the interview, tape verbatim will be transcribed, noting pauses, changes in tone, laughter, comments, and affirmative “noises.” In addition, the length of the interview and amount of time required to transcribe will also be noted at the end of the transcript  The interview will be conducted by a team of two researchers. One person will ask the questions, and the other will record the responses, both in writing and by an audio recorder.  **General instructions**   - **Welcome the participant** - **Overview of the topic:** The overall aim of the study is to demonstrate that in already vulnerable populations further affected and displaced by climate change-related crises such as mass flooding, mental health screening and referral can be successfully implemented by community health workers, along with community-level education/awareness sessions and other activities designed to build community, household, and individual-level resilience to the effects of climate change, including the mental health effects. - **Purpose of the KII:** The purpose of KII is to explore disaster management official’s views regarding the impact of climate change on mental health and ways to tackle it. More importantly, their views on disaster management for flood-affected communities will also be noted. Their available resources and capacity to manage flood-related disasters will be explored, as well as what challenges they face from the community. Their perspective on lived experiences in the community will be noted, along with how community resiliency can help disaster management officials better cope with flooding/climate change events.   **Ground rules of KII:**   - Please talk in a loud voice. - Kindly feel free not to respond to questions that you cannot relate to and feel uncomfortable answering. - Please ask questions/clarification as they come up. |
| --- |

KII session No: ________________

**PARTICIPANT’S INFORMATION: to be filled by interviewer**

| Name of Disaster Management official |  |
| --- | --- |
| Gender |  |
| Age |  |
| Designation |  |
| Place of work or institution |  |
| Work experience |  |
| Education Level |  |
| Qualification |  |
| Contact details |  |

| Name of Interviewer |  |
| --- | --- |
| Name of note taker |  |
| Duration of interview | Begin End |
| Date of Interview | DD / MM/ YY |

| **S. No.** | **Lead** | **Comments** |
| --- | --- | --- |
| **Disaster management (DM) official’s perceptions regarding climate change (floods) and its impact on mental health in the community** | | |
|  | What do you understand by ‘mental health’?  Probe:   - What is the prevalence of mental health issues in the community under consideration? - What are available mental health resources? |  |
|  | What comes to your mind when you hear the term ‘climate change’?  Probes:   - How do you think climate change happens/what causes climate change? - Is there any link between mental health and climate change? If yes, please elaborate. |  |
|  | What disaster management services are available to the community?  Probes:   - What additional measures were taken during floods? - What are National Adaptation Plan recommendations? - Are they implemented? How? - Are LHWs/LHS/BHU staff part of the implementation plan? |  |
|  | What impact did the floods of 2022 have on the mental health of the community?  Probes:   - Effect on community WRAs - Effects of displacement |  |
|  | What does the community think about the provision of disaster management services, particularly in relation to floods?  Probes:   - What services, helplines, and shelter homes are provided? - Capacity of Govt. disaster management system - How well-received are these services in the community? |  |
|  | Can you describe the lived experiences of the communities during the 2022 floods?  Probes:  - What problems did they face during and in the aftermath of the floods?  - What problems did they face during the rehabilitation phase?  - What losses did they incur and how did they deal with it? |  |
|  | How does your department deal with the mental health impact of the floods?  Probe:   - Available resources/referral facilities to refer community which is facing problems |  |
| **Perceptions regarding building community resilience to climate change in flood-affected areas** | | |
|  | What comes to your mind when you hear ‘community resilience’?  Probes:   - Previous measures taken to build community resilience - What communities can do as a whole to become better equipped to face natural disasters like floods? |  |
|  | What challenges do you face dealing with natural disasters?  Probes:   - Available resources, - Infrastructure, - Impact of climate change on disaster management strategies (are your protocols/work affected by climate events like floods, etc?) - Is the community mentally equipped during natural disasters to engage with disaster management authorities? |  |
|  | Importance of conducting group mental health and resilience-building awareness in community  Probes:   - How will this impact disaster management officials’ work? - How effective could this be in enhancing disaster preparedness in the community? - any previous experience in building community resilience against floods? |  |
|  | What do you think about having frontline workers building resilience for WRAs within the communities? |  |

We have reached the end of our interview. Thank you for your participation.
