# Supplementary material for: Community-based mental health screening & referral for flood-affected women in rural Pakistan: an intervention feasibility study protocol
Source: BMJ Open. 2025 Oct 23;15(10):e104759. doi: 10.1136/bmjopen-2025-104759 (PMC12551463; doi:10.1136/bmjopen-2025-104759)
Supplement: online supplemental file 8 [file bmjopen-15-10-s008.docx]

**Community-Based Mental Health Screening & Referral for Flood-Affected Women in Dadu: A Feasibility Study**

**Qualitative Component**

**Interview Guide for Post-Intervention for Policy Makers**

| **Guidelines for post-intervention Key Informant Interviews (KII) with Policymakers**.  One semi-structured questionnaire will be used for each participant who has consented.  **Consent**: Written consent form will be signed by each participant before commencing each key informant interview.  **Duration**: 30 minutes will be allocated, or it can be extended until the point of saturation.  **Mode of recording**: A tape recorder will be used for recording each key informant interview. In addition, written notes will also be taken during the interview.  **Place for interview**: Office of the participant/AKU/online over zoom whatever is feasible.  **Transcription**: Following the interview, tape verbatim will be transcribed, noting pauses, changes in tone, laughter, comments, and affirmative “noises.” In addition, the length of the interview and amount of time required to transcribe will also be noted at the end of the transcript, so that other key informant interviews can be modified or implemented accordingly. The interview will be conducted by a team of two researchers. One person will ask the questions, and the other will record the responses, both in writing and with an audio recorder.  **General instructions**   - **Welcome the participant** - **Overview of the topic:** The overall aim of the study is to demonstrate that in already vulnerable populations further affected and displaced by climate change-related crises such as mass flooding, mental health screening and referral can be successfully implemented by community health workers, along with community-level education/awareness sessions and other activities designed to build community, household, and individual-level resilience to the effects of climate change, including the mental health effects. - **Purpose of the KII:** The purpose of KII is to explore policymaker’s views regarding uptake of intervention and barriers and facilitators to implementation roll out.   **Ground rules of KII**   - Please talk in a loud voice. - Kindly feel free not to respond to questions that you cannot relate to and feel uncomfortable answering. - Please ask questions/clarification as they come up. |
| --- |

KII session No: ________________

**PARTICIPANT’S INFORMATION:** To be filled by participant

| Name of Policymaker |  |
| --- | --- |
| Gender |  |
| Age |  |
| Designation |  |
| Place of work or institution |  |
| Work experience |  |
| Education Level |  |
| Qualification |  |
| Contact details |  |

To be filled by interviewer

| Name of Interviewer |  |
| --- | --- |
| Name of recorder/volunteer |  |
| Duration of interview | Begin  End |
| Date of Interview | DD / MM/ YY |

| **S. No.** | **Lead** | **Comments** |
| --- | --- | --- |
| **Uptake of Intervention** | | |
|  | What are your views regarding LHWs delivering mental health screening and referral in the community?  Probes:   - Was the LHW-P equipped to undertake intervention? - Coordination between LHWs, LHSs and LHW-P program office - What do you think about the LHW’s time and willingness for this intervention? |  |
|  | What are your views regarding LHWs delivering group mental health awareness and resilience-building sessions?  Probes:   - Was the LHW-P equipped to deliver the group session? - Community acceptance of group session - Problems faced by LHWs and LHSs during group session - Did the session help in raising mental health and community resilience awareness among community participants? - Recommendations for future |  |
|  | What are your views regarding referral facility (BHU/RHC) conducting mental health screening, counselling and handling data management of referred cases?  Probes:   - Was there sufficient coordination between LHW-P and referral facility staff? - Was communicating information about referral cases easy for LHWs/LHSs to facility staff? - Any other challenges faced during implementation of referral pathway |  |
|  | What was the benefit of intervention for mental health service delivery and increasing community resilience in the District?  Probes:   - Does this intervention help in raising mental health awareness and community resilience to climate change (especially flood-related resiliency)? - Were the intended audience (WRA) the best recipients (as they are affected most by floods) or should the intervention be scaled up for the general adult population? |  |
| **Barriers in implementation roll out** | | |
|  | What were the factors that acted as barriers during implementation roll out?  Probes:   - LHWs’ time and capacity for service delivery, workload issues - Project flow – issues in adhering to timelines (time between LHW screening and reporting to LHS, then LHS confirming referral cases, LHW visiting referral case on next routine visit – did this process work smoothly?) - Is this adding unnecessary steps in primary healthcare delivery? |  |
|  | Suggestions for improvement and sustainability |  |
| **Facilitators in implementation roll out** | | |
|  | What were the factors that facilitated implementation roll out?  Probes:   - Role of LHS’s supervision - Role of LHW’s acceptability in community - Role of referral facility in making mental health screening and counselling more accessible - Customized training to all cadres of health workers to facilitate project flow |  |
|  | What was the feasibility of LHW-P facilitating community-based screening and referral and conducting group mental health and community resilience awareness sessions?  Probes:   - Was it successful? - Recommendations for scaling up |  |

We have reached the end of our interview. Thank you for your participation. Do you have any further feedback?
