# Supplementary material for: Community-based mental health screening & referral for flood-affected women in rural Pakistan: an intervention feasibility study protocol
Source: BMJ Open. 2025 Oct 23;15(10):e104759. doi: 10.1136/bmjopen-2025-104759 (PMC12551463; doi:10.1136/bmjopen-2025-104759)
Supplement: online supplemental file 9 [file bmjopen-15-10-s009.docx]

**Community-Based Mental Health Screening & Referral for Flood-Affected Women in Dadu: A Feasibility Study**

**Qualitative Component**

**Interview Guide for Post-Intervention for Referral Facility Staff**

| **Guidelines for post-intervention Key Informant Interviews (KII) with Referral Facility Staff (RF)** who participated in mental health screening, counselling, and management of referred community participants (women of reproductive age) during intervention roll out.  One semi-structured questionnaire will be used for each participant who has consented.  **Consent**: Written consent form will be signed by each participant before commencing each key informant interview.  **Duration**: 30 minutes will be allocated, or it can be extended until the point of saturation.  **Mode of recording**: A tape recorder will be used for recording each key informant interview. In addition, written notes will also be taken during the interview.  **Place for interview**: Any suitable place in the public sector health facility/ Department of Health/Zoom will be chosen as per the participant’s comfort.  **Transcription**: Following the interview, tape verbatim will be transcribed, noting pauses, changes in tone, laughter, comments, and affirmative “noises.” In addition, the length of the interview and amount of time required to transcribe will also be noted at the end of the transcript, so that other key informant interviews can be modified or implemented accordingly. The interview will be conducted by a team of two researchers. One person will ask the questions, and the other will record the responses, both in writing and with an audio recorder.  **General instructions**   - **Welcome the participant** - **Overview of the topic:** The overall aim of the study is to demonstrate that in already vulnerable populations further affected and displaced by climate change-related crises such as mass flooding, mental health screening and referral can be successfully implemented by community health workers, along with community-level education/awareness sessions and other activities designed to build community, household, and individual-level resilience to the effects of climate change, including the mental health effects. - **Purpose of the KII:** The purpose of KII is to explore referral facility staff’s views regarding uptake of intervention and barriers and facilitators to implementation roll out.   **Ground rules of KII**   - Please talk in a loud voice. - Kindly feel free not to respond to questions that you cannot relate to and feel uncomfortable answering. - Please ask questions/clarification as they come up. |
| --- |

KII session No: ________________

**PARTICIPANT’S INFORMATION:** To be filled by participant

| Name of RF staff |  |
| --- | --- |
| Gender |  |
| Age |  |
| Designation |  |
| Place of work or institution |  |
| Work experience |  |
| Education Level |  |
| Qualification |  |
| Contact details |  |
| How many screen-positive WRAs were referred to you? |  |

To be filled by interviewer

| Name of Interviewer |  |
| --- | --- |
| Name of recorder/volunteer |  |
| Duration of interview | Begin  End |
| Date of Interview | DD / MM/ YY |

| **S. No.** | **Lead** | | **Comments** |
| --- | --- | --- | --- |
| **Uptake of Intervention** | | | |
|  | What do you understand by ‘mental health’?  Probes:   - What is ‘good’ mental health vs ‘poor’?   How do you judge someone’s mental health? | |  |
|  | How was your experience with training on mental health screening, counselling, and management?  Probes:   - Was training useful? - Did it improve your skills in dealing with mental health referrals from the community? | |  |
|  | How was your experience with dealing with referred WRAs?  Probes:   - Did referral cases increase with LHW screening and referral intervention? - How effective was mental health screening and counselling with referral cases? - Referred participant’s eagerness/reluctancy to receive counselling - How many referred cases showed up to referral facility? What was the general trend? - Did referred participants find it easy to come to the facility? | |  |
|  | What are your views regarding LHWs doing mental health screening and referral?  Probes:   - Was LHW screening effective in referring cases with mental health issues? - What could be done to improve their screening accuracy? | |  |
|  | What are your views regarding LHWs delivering group mental health awareness and resilience sessions with referral cases before sending to referral facility?  Probes:   - Was the group session effective in increasing mental health awareness of participants? - Did attending the group session make them less anxious and depressed? What was their perspective? | |  |
|  | What are your views regarding RF staff conducting mental health screening, counselling and managing data  Probes:   - Was record-keeping and maintaining logs for referred cases easy to do? - How easy was it to do counselling for referred cases? - How many participants needed to be referred to tertiary care facilities for pharmacological intervention? | |  |
| **Barriers to implementation roll out** | | | |
|  | Were there any barriers to implementation roll out?  Probes:   - Time, resource constraints - WRA willingness to come to facility - Infrastructure availability in RF to accommodate referral cases - Capacity to send severe cases to tertiary care - Troubleshooting/dealing with adverse events | |  |
|  | Suggestions for improvement and sustainability | |  |
| **Facilitators to implementation roll out** | | | |
|  | What were the factors that facilitated implementation roll out?  Probes:   - Training by study team - Allocation of resources by LHW-P - Allocation of resources by Health Dept |  | |
|  | What was the feasibility of referral facility staff doing mental health screening, counselling, and managing referral patient data?  Probes:   - Was it successful? - Recommendations for future |  | |

We have reached the end of our interview. Thank you for your participation. Do you have any further feedback?
