# Supplementary material for: Community-based mental health screening & referral for flood-affected women in rural Pakistan: an intervention feasibility study protocol
Source: BMJ Open. 2025 Oct 23;15(10):e104759. doi: 10.1136/bmjopen-2025-104759 (PMC12551463; doi:10.1136/bmjopen-2025-104759)
Supplement: online supplemental file 10 [file bmjopen-15-10-s010.docx]

**Community-Based Mental Health Screening & Referral for Flood-Affected Women in Dadu: A Feasibility Study**

**Qualitative Component**

**FGD Guide for Post-Intervention for LHSs**

| **Guidelines for post-intervention Focus Group Discussion (FGD) with Lady Health Supervisors (LHSs)** who took part in the intervention delivery  **Consent**: Written informed consent will be signed by each participant before commencing the FGD.  **Duration**: Nearly 45 minutes will be allocated, or it can be extended until the point of saturation.  **Mode of recording**: Tape recorder will be used for recording. In addition, written notes will also be taken.  **Place for FGD**: LHW-P office where participants feel comfortable, and their privacy will be ensured.  **Transcription**: Following the completion of each discussion, tape verbatim will be transcribed, noting pauses, changes in tone, laughter, and moderator’s questions, comments, and affirmative “noises.” In addition, length of FGD and amount of time required to transcribe will also be noted at the end of transcript, so that other FGDs can be modified or implemented accordingly.  FGD will be conducted by a team of two researchers. One person will moderate the session, and the other will record the responses, both in writing and by audio recorder.  **General instructions**   - **Welcome the participants.** - **Overview of the topic:** The overall aim of the study is to demonstrate that in already vulnerable populations further affected and displaced by climate change-related crises such as mass flooding, mental health screening and referral can be successfully implemented by community health workers, along with community-level education/awareness sessions and other activities designed to build community, household, and individual-level resilience to the effects of climate change, including the mental health effects. - **Purpose of the FGD:** The purpose of FGD is to explore LHSs’ experience regarding uptake of intervention and the barriers and facilitators experienced during implementation roll out.   **Ground rules of FGD**   - Please talk in a loud voice. - Kindly feel free not to respond to questions that you cannot relate to and feel uncomfortable answering. - Please ask questions/clarification as they come up. - Kindly respect each other’s opinion |
| --- |

FGD session No: ________________

**Session attendance information sheet** (To be filled by participants)

| **S. No.** | **Name of LHS** | **Age (yrs)** | **Catchment area/taluka** | **Work experience (yrs)** | **Contact details.** | **Education level (matric, intermediate, university degree, post-graduate qualification)** |
| --- | --- | --- | --- | --- | --- | --- |
|  |  |  |  |  |  |  |
|  |  |  |  |  |  |  |
|  |  |  |  |  |  |  |
|  |  |  |  |  |  |  |
|  |  |  |  |  |  |  |
|  |  |  |  |  |  |  |
|  | (To be filled by moderator)  **Date of FGD: __/__/____ Duration of FGD: ________**  **UC Name:**  **Village Name:**  **Place of FGD Begin - __: __**  **Name of moderator: End - __: __**  **Name of note taker:** | | | | | |

| **S. No.** | **Lead** | **Comments** |
| --- | --- | --- |
| **Intervention uptake** | | |
|  | What do you understand by ‘mental health’?  Probes:   - What sources do you get information on mental health from? - How do you judge someone’s mental health?   What is ‘good’ mental health vs ‘poor’? |  |
|  | How well did the LHWs receive the mental health screening and referral services?  Probes:   - How was your experience during intervention roll out - What are your views on supportive supervision for LHWs during screening and referral? - What was the ease of communication between LHW and LHS for referral cases/screening scores, - Were the WRAs satisfied or unsatisfied? Why? |  |
|  | How did the flood-affected WRAs receive the mental health screening and referral services?  Probes:   - Community satisfaction/dissatisfaction to receive mental health screening and referral by LHWs |  |
|  | How well did you receive this intervention?  Probes:   - LHWs confidence in using brief screening tools, - LHWs time allocation for this tool during their routine visits, - LHWs satisfaction of delivering screening, - Response to LHWs when referring community WRAs |  |
|  | How easy do you think the brief and rapid screening tools for assessment of mental health were?  Probe:   - Were the tools easily deployed? Please elaborate on why. |  |
|  | What challenges did you face while supervising and tracking referral cases from LHWs?  Probes:   - Data Collection and Management - Availability of WRAs - Issues of transport/access - Any other challenges |  |
|  | How effective were the rapid screening tools?  Probes:   - Did the tools lead to reliable assessments? Please elaborate on why. |  |
|  | How effective was using danger signs for referrals for specialist care? |  |
|  | How useful were the group mental health awareness and resilience-building sessions delivered by LHWs in the community?  Probes:   - Was there an effective utilization of LHWs’ time? - Was there significant community engagement? - Was there any usefulness of the sessions in reducing mental health symptoms? - Was there any usefulness of the sessions in increasing community resilience to flooding caused in part due to climate change? - What was the impact of the sessions in increasing awareness about dealing with natural disasters? |  |
|  | How useful was it to refer participants to BHUs/RHCs for specialist care?  Probes:   - Did referral to specialized care facility increase because of LHWs’ screening? - Were referral cases catered to sufficiently at referral facility? - What factors contributed to acceptance/rejection of being referred in community WRAs? |  |
| **Barriers to Implementation** | | |
|  | What barriers did you face during the intervention roll out?   - Problems faced between LHW/LHS coordination - Problems faced between LHW and referral cases - Problems faced by LHSs’ during LHWs’ group session delivery - Any other problems? |  |
|  | Suggestions for improvement and sustainability |  |
| **Facilitators to Implementation** | | |
|  | What do you think about the role played by the facilitators during the intervention roll out?   - Role of supportive supervision - Role of referral facility staff - Any other facilitators? |  |

We have reached the end of our interview. Do you have any additional suggestions for LHWs conducting mental health screening and referral in flood-affected areas?
