# Supplementary material for: Community-based mental health screening & referral for flood-affected women in rural Pakistan: an intervention feasibility study protocol
Source: BMJ Open. 2025 Oct 23;15(10):e104759. doi: 10.1136/bmjopen-2025-104759 (PMC12551463; doi:10.1136/bmjopen-2025-104759)
Supplement: online supplemental file 11 [file bmjopen-15-10-s011.docx]

**Community-Based Mental Health Screening & Referral for Flood-Affected Women in Dadu: A Feasibility Study**

**Qualitative Component**

**Interview Guide for Pre-Intervention for Referral Facility Staff**

| **Guidelines for Formative Phase Key Informant Interviews (KII) with Referral Facility Staff (RF)**  One semi-structured questionnaire will be used for each participant who has consented.  **Consent**: Written informed consent will be signed by each participant before commencing each KII.  **Duration**: 30 minutes will be allocated, or it can be extended until the point of saturation.  **Mode of recording**: A tape recorder will be used for recording the key informant interview. In addition, written notes will also be taken during the interview.    **Place for FGD**: BHU/RHC (referral facility) office where participants feel comfortable, and their privacy will be ensured.  **Transcription**: Following the interview, tape verbatim will be transcribed, noting pauses, changes in tone, laughter, comments, and affirmative “noises.” In addition, the length of the interview and amount of time required to transcribe will also be noted at the end of the transcript  The interview will be conducted by a team of two. One person will ask the questions, and the other will record the responses, both in writing and by an audio recorder.  **General instructions**     - **Welcome the participants** - **Overview of the topic:** The overall aim of the study is to demonstrate that in already vulnerable populations further affected and displaced by climate change-related crises such as mass flooding, mental health screening and referral can be successfully implemented by community health workers, along with community-level education/awareness sessions and other activities designed to build community, household, and individual-level resilience to the effects of climate change, including the mental health effects.      - **Purpose of the FGD:** The purpose of the KII is to explore referral facility staff’s views regarding the burden of mental illness in their community and ways to tackle this burden. This FGD will take referral facility staff’s opinion on their capacity and readiness to undertake capacity building training for diagnostic and management skills in mental health. Their perspective on readiness as a referral facility for mental health diagnostic, counselling and management services will be explored.   **Ground rules of KII**   - Please talk in a loud voice. - Kindly feel free not to respond to questions that you cannot relate to and feel uncomfortable answering. - Please ask questions/clarification as they come up. |
| --- |

KII session No:

**PARTICIPANT’S INFORMATION: to be filled by interviewer**

| Name of RF staff |  |
| --- | --- |
| Gender |  |
| Age |  |
| Designation |  |
| Place of work or institution |  |
| Work experience |  |
| Education Level |  |
| Qualification |  |
| Contact details |  |

| Name of Interviewer |  |
| --- | --- |
| Name of note taker |  |
| Duration of interview | Begin End |
| Date of Interview | DD / MM/ YY |

| **S. No.** | **Lead** | **COMMENTS** |
| --- | --- | --- |
| **Mental health disease burden and impact of climate change on mental health** | | |
|  | What do you understand by ‘mental health’?  Probes:   - What sources do you get information on mental health from? - What is ‘good’ mental health vs ‘poor’? - How do you judge someone’s mental health? |  |
|  | How is the overall mental health of the community?  Probes:   - How common are mental health issues in the community? - Are WRA (women of reproductive age) affected and how? |  |
|  | Are you aware of any available mental health services within your community?  Probe:   - What are the available resources? - How accessible are these resources? - How is the experience of others like using these services? - Do they face any stigma and/or discrimination while using such services? |  |
|  | Do you think LHWs can effectively deliver mental health services during home visits?  Probe:   - Human resource, - time, - willingness. - What capability do you think is required for effective delivery? - What are the factors for willingness or reluctance to take on mental health service delivery? |  |
|  | How likely will the community make use of mental health services delivered by LHWs?  Probe:   - What barriers exist to the uptake of such services? - What facilitators exist for the uptake of such services? - Is the RF staff equipped to deal with any referrals made by LHWs to their facility? |  |
|  | How did the community react to the floods?  Probe:   - Difficulties faced by WRAs - Were there any protective/preventative measures in place by the community? - How did the community handle displacement because of the floods? |  |
|  | What impact did the floods of 2022 have on the mental health of the community?  Probe:   - What were communities most worried about when the floods hit? - What other emotions/feelings did the victims experience during the floods and in their aftermath? - Please describe the lived experiences of those affected by the floods. If you have any examples of community resilience, please share those too. |  |
| **Readiness assessment of referral facilities for mental health screening, counselling, and management services** | | |
|  | How comfortable would you be in receiving training for mental health screening, counselling, and data management skills?  Probe:   - Human resource, - time, - willingness, - space - What impact would this training have on your existing responsibilities and workflows? |  |
|  | Do you think that the management would be willing to allow you to receive such training?  Probe:   - Resource allocation, - political willingness/reservations |  |
|  | Is the current infrastructure at your facility equipped to facilitate those with mental health problems?  Probe:   - Is facility equipped to include space for counselling?, - Will space be secluded (as referral WRA may require privacy during counselling)? - What other infrastructure do you think is required to effectively provide services to those suffering from mental health problems? |  |
|  | How would you manage record-keeping for the referrals made by LHWs?  Probe:   - Maintain patient logs of referred WRAs - Records of patient’s screening - Number of counselling sessions - Referrals to tertiary care hospital for pharmacological intervention for severe cases |  |
| **Feasibility of strategy** | | |
|  | What potential challenges do you think this intervention would face and what solutions do you think can be implemented?  Probe:   - What difficulties can BHU/RHC face in administering mental health screening, counselling and management services? - What role could facility management play in overcoming these difficulties? |  |

We have reached the end of our interview. Thank you for your participation.
