# Supplementary material for: Community-based mental health screening & referral for flood-affected women in rural Pakistan: an intervention feasibility study protocol
Source: BMJ Open. 2025 Oct 23;15(10):e104759. doi: 10.1136/bmjopen-2025-104759 (PMC12551463; doi:10.1136/bmjopen-2025-104759)
Supplement: online supplemental file 12 [file bmjopen-15-10-s012.docx]

**Community-Based Mental Health Screening & Referral for Flood-Affected Women in Dadu: A Feasibility Study**

**Qualitative Component**

**Interview Guide for Pre-Intervention for Policy Makers**

| **Guidelines for Formative phase Key Informant Interviews with Policy Makers (PM)**  One semi-structured questionnaire will be used for the participant who has consented.  **Consent**: Written consent form will be signed by the participant before commencing each key informant interview.  **Duration**: 30 minutes will be allocated, or it can be extended until the point of saturation.  **Mode of recording**: A tape recorder will be used for recording the key informant interview. In addition, written notes will also be taken during the interview.  **Place for interview**: Office of the participant/AKU/online over zoom whatever is feasible.    **Transcription**: Following the interview, tape verbatim will be transcribed, noting pauses, changes in tone, laughter, comments, and affirmative “noises.” In addition, the length of the interview and amount of time required to transcribe will also be noted at the end of the transcript  The interview will be conducted by a team of two. One person will ask the questions, and the other will record the responses, both in writing and by an audio recorder.  **General instructions**   - **Welcome the participant** - **Overview of the topic:** The overall aim of the study is to demonstrate that in already vulnerable populations further affected and displaced by climate change-related crises such as mass flooding, mental health screening and referral can be successfully implemented by community health workers, along with community-level education/awareness sessions and other activities designed to build community, household, and individual-level resilience to the effects of climate change, including the mental health effects. - **Purpose of the KII:** The purpose of KII is to explore policy makers’ views regarding the burden of mental illness in the community and ways to tackle it. The views of these policy makers and administrators on disaster management for flood-affected communities will also be noted. Moreover, their opinion regarding the capacity of LHW-P to provide home-based mental health screening and referral services through the LHWs, and supervision by LHSs will also be taken. Perceptions of policymakers about the ability of LHWs to provide group mental health awareness and resilience building sessions in the community will also be explored. Policy makers will also highlight their views about the preparedness and readiness of referral BHU/RHC facility and staff to provide appropriate screening and counselling services to the referred women of reproductive age (WRAs).   **Ground rules of KII:**   - Please talk in a loud voice. - Kindly feel free not to respond to questions that you cannot relate to and feel uncomfortable answering. - Please ask questions/clarification as they come up. |
| --- |

KII session No: ________________

**PARTICIPANT’S INFORMATION: to be filled by interviewer**

| Name of Policy maker/implementer |  |
| --- | --- |
| Gender |  |
| Age |  |
| Designation |  |
| Place of work or institution |  |
| Work experience |  |
| Education Level |  |
| Qualification |  |
| Contact details |  |

| Name of Interviewer |  |
| --- | --- |
| Name of note taker |  |
| Duration of interview | Begin End |
| Date of Interview | DD / MM/ YY |

| **S. No.** | **Lead** | **Comments** |
| --- | --- | --- |
| **Policymaker’s perceptions regarding mental health disease burden, mental health services** | | |
|  | Could you tell us how ‘mental health’ is understood in a disaster management context?  Probes:   - How common are mental health issues in the community? - Are WRAs affected and how? |  |
|  | Are you aware of any available mental health services within communities?  Probes:   - What resources are made available to communities? - How accessible are these resources? |  |
|  | In your opinion, how likely is it that the community will readily accept the provision of mental health services by LHWs?  Probe:   - Community factors leading to acceptance or rejection to receive mental health services from LHWs |  |
| **Policy maker’s perceptions regarding mental health impact of climate change, and disaster management services** | | |
|  | What impact did the floods of 2022 have on the mental health of the community?  Probes:   - Effect on community WRA, - displacement, temporary shelters - mental distress |  |
|  | What does the community think about the provision of disaster management services, particularly in relation to floods?  Probes:   - What services, helplines, shelter homes are provided? - Capacity of Govt. disaster management system - How well-received are these services in the community? |  |
|  | Can you describe the lived experiences of the communities during the 2022 floods?  Probes:   - What problems did they face during and in the aftermath of the floods? - What problems did they face during the rehabilitation phase? - What losses did they incur and how did they deal with it? |  |
|  | How do your policies deal with the mental health impact of the floods?  Probes:   - Challenges in dealing with natural disasters - Available resources/referral facilities to refer community which is facing problems |  |
| **Acceptability and appropriateness of LHWs for mental health screening and referral, and delivering group mental health awareness and resilience-building sessions** | | |
|  | Do you think that LHWs can effectively provide mental health screening and referral services to the community?  Probes:   - LHW/LHS skill set, human resource, time, willingness. - LHW skills and competency to administer short screening tools - LHW skills to successfully refer WRAs to BHU/RHC level - Acceptability of this mode of service delivery, professional factors leading to acceptance or rejection by LHSs and LHWs - Opinion regarding LHSs’ supportive supervision during this process |  |
|  | How effectively can LHWs deliver group mental health awareness and resilience-building sessions to referred community WRAs?  Probes:   - LHW skillset, time, willingness - LHWs’ response to capacity building training - Community WRA’s response to group sessions - Resources available (space, time, availability of community participants) - What could be the intended effect of such training? - Opinion regarding LHSs’ supportive supervision during this process |  |
| **Readiness assessment of referral facility for mental health screening, counselling and management services** | | |
|  | How ready are BHU/RHC staff for training in mental health screening, counselling, and record keeping skills?  Probes:   - Are there available human resources, time available, willingness of the staff, and space within facilities? |  |
|  | How willing is the Department of Health and the facility management to integrate this intervention in their processes? |  |
|  | How capable is the infrastructure at the referral facility?  Probes:   - Is the facility equipped to include space for counselling? - Will space be secluded (as referral WRA may require privacy during counselling) - Is there any other infrastructure that can enhance the effectiveness of this intervention? |  |
|  | How effectively do you think the facility staff can keep and manage records?  Probes:   - Maintain patient logs of referred WRAs - Records of diagnostic assessments performed - Number of counselling sessions - Referrals to tertiary care hospital for pharmacological intervention for severe cases |  |
| **Feasibility of strategy** | | |
|  | What are the potential challenges this intervention will face and what solutions can be implemented?  Probes:   - What difficulties can LHWs face from the community? - What difficulty could LHSs face during supervision? - What difficulties can referral facility staff face? - What role can policy makers play in overcoming these difficulties? |  |
|  | Input on how policy makers could facilitate the process of mental health service provision through LHW-P  Probes:   - Funding, - Modifying LHW-P job description, - Capacity building trainings, - Community sensitization (mental health awareness). - Management of facility staff capacity and infrastructure |  |

We have reached the end of our interview. Thank you for your participation.
