# Supplementary material for: Community-based mental health screening & referral for flood-affected women in rural Pakistan: an intervention feasibility study protocol
Source: BMJ Open. 2025 Oct 23;15(10):e104759. doi: 10.1136/bmjopen-2025-104759 (PMC12551463; doi:10.1136/bmjopen-2025-104759)
Supplement: Supplementary file 13 [file bmjopen-2025-104759.reviewer_comments.pdf]

PEER REVIEW HISTORY

BMJ Open publishes all reviews undertaken for accepted manuscripts. Reviewers are asked to complete a checklist review form and are provided with free text boxes to elaborate on their assessment. These free text comments are reproduced below.

ARTICLE DETAILS

Title (Provisional)

Community-Based Mental Health Screening & Referral for Flood-Affected Women in Rural Pakistan: An Intervention Feasibility Study Protocol

Authors

Das, Jai K.; Gaffey, M F; Ansari, Zoya Navid; Mirani, Mushtaque; Tabassum, Farhana; Siddiqui, Amna; Rabbani, Fauziah; Rizvi, Arjumand; Ahmed, Imran; Khan, Murad; Bhutta, Zulfiqar Ahmed

---

VERSION 1 - REVIEW

**Reviewer**	**1**
**Name**	**Azmat, Syed Khurram**
**Affiliation**	**Jinnah Sindh Medical University, AAPNA - Institute of Public Health**
**Date**	**10-Aug-2025**
**COI**	**None**

The study tackles an important and largely unexplored gap by integrating community-based mental health services into primary healthcare within disaster-affected rural areas of Pakistan. While the design is well-aligned with the local context and informed by formative research, the use of a quasi-experimental approach reduces internal validity. There are also operational challenges in implementing the intervention, and the manuscript’s reporting could be improved through more explicit theoretical grounding and methodological clarity. Strengthening these aspects would enhance both reliability and the potential for scaling the findings.

Overall design: The study uses a quasi-experimental design with no randomisation, raising concerns due to the risk of baseline differences and selection bias (based on purposive selection of UCs for intervention and control groups). To strengthen internal validity, the authors may consider running matched cases or, ideally, employing cluster randomisation.

The manuscript mentions that “the formative phase evaluated….”, has this already been completed?

While demographic adjustments are planned, the other variables, such as environmental and social (e.g., access to NGO services, seasonal stressors), are not factored.

Define primary and secondary outcome variables, preferably with citations further clarifying them by separating feasibility metrics from the effectiveness outcome variables. Also, without baseline outcome data, the utility of DID analysis is limited. A period of four months may be too short to detect sustained mental health improvements or resilience changes. Also, it would be the authors' responsibility to run a sensitivity analysis, which will help them test the robustness against potential confounders.

The LHW cadre is mostly middle-level educated, so is there a strong rationale and credible evidence that they can be trained for screening the "Generalised Anxiety Disorder scale"? Additionally, the high workload of LHWs (screening ~ 120 WRA, running sessions, etc.) may lead to professional saturation when they perform their parallel routine work. In addition, LHW screening may pose a risk of labelling communities with high stigma towards mental health, so what are the authors' plans to counter this narrative? Also, limited transport support poses a threat to loss of follow-up in severe cases.

Background: Strongly focused on climate change and disaster context, but less emphasis on existing mental health service delivery challenges in Sindh (e.g., past PHC integration efforts).

The cultural adaptation, translation, and validation tools, such as GAD-2/PHQ-2 for flood-affected rural women are not detailed.

The link between formative insights and the selected strategies seems weak – can you describe more about the integration of qualitative data into the intervention design?

Interestingly, there is no theory of change or a conceptual framework presented, which seems a key for such studies involving human behaviour and mental health. Unfortunately, this limits the understanding of how screening, counselling, and resilience-building are expected to influence mental health outcomes.

If possible, the authors are requested to provide information on the costing of such a strategy or the cost-effectiveness benefit of this study.

The authors have discussed the distress protocol; however, operational details, including the timeline of action, delivery of more information, and referral tracking, appear to be lacking.

The area and target population focused have a low level of literacy; therefore, written consent seems challenging. So, what are the alternatives?

VERSION 1 - AUTHOR RESPONSE

**Reviewer**	

The study tackles an important and largely unexplored gap by integrating community-based mental health services into primary healthcare within disaster-affected rural areas of Pakistan. While the design is well-aligned with the local context and informed by formative research, the use of a quasi-experimental approach reduces internal validity. There are also operational challenges in implementing the intervention, and the manuscript's reporting could be improved through more explicit theoretical grounding and methodological clarity. Strengthening these aspects would enhance both reliability and the potential for scaling the findings.	We would like to thank the reviewer for the thorough review and valuable input. We have reviewed the comments and revised the manuscript accordingly and responded to each specific comment below.
Overall design: The study uses a quasi-experimental design with no randomisation, raising concerns due to the risk of baseline differences and selection bias (based on purposive selection of UCs for intervention and control groups). To strengthen internal validity, the authors may consider running matched cases or, ideally, employing cluster randomisation.	The comment is well noted and an RCT would be a perfect design for assessing the effectiveness of any intervention. But the primary objective of this study is to first assess the feasibility of such an intervention in a primary health system of a LMIC and to assess if the system is ready to take on this specific intervention. Hence, we have purposively employed a quasi-experimental design because of the focus on feasibility of the intervention rather than its effectiveness and even in this we would have baseline data to compare the groups. If this study proves the feasibility, then as a next step, we would like to proceed with the effectiveness trial and choose cluster RCT. We hope that this answers the query adequately.
The manuscript mentions that "the formative phase evaluated….", has this already been completed?	Yes, the formative phase was conducted beforehand to better inform the design utilized in the study. We have now mentioned the timelines and also added key findings from the formative phase on page 6-7.
While demographic adjustments are planned, the other variables, such as environmental and social (e.g., access to NGO services, seasonal stressors), are not factored.	Thank you for this feedback and we will keep a note of this when planning on the next phase RCT where we will assess its effectiveness. But we would also like to highlight that as this study is being conducted in a single district, we do not anticipate much variance in access to NGO services and other seasonal stressors.
Define primary and secondary outcome variables, preferably with citations further clarifying them by separating feasibility metrics from the effectiveness outcome variables. Also, without baseline outcome data, the utility of DID analysis is limited. A period of four months may be too short to detect sustained mental health improvements or resilience changes. Also, it would be the authors' responsibility to run a sensitivity analysis, which will help them test the robustness against potential confounders.	This research study will focus on the feasibility of integrating mental health services within the existing healthcare system. It is a pilot project evaluating feasibility rather than effectiveness (which is a secondary objective here), which will be investigated in a follow-up trial planned on a larger scale.<br><br>But we are doing a baseline assessment (page 10) even in this study and collecting information on all outcome variables at both baseline and endline.<br><br>The period of four months may be too short, and that is why this is a secondary outcome, and the results would better inform the next steps of the trial.

The LHW cadre is mostly middle-level educated, so is there a strong rationale and credible evidence that they can be trained for screening the "Generalised Anxiety Disorder scale"? Additionally, the high workload of LHWs (screening ~ 120 WRA, running sessions, etc.) may lead to professional saturation when they perform their parallel routine work. In addition, LHW screening may pose a risk of labelling communities with high stigma towards mental health, so what are the authors' plans to counter this narrative? Also, limited transport support poses a threat to loss of follow-up in severe cases.	Women living in these rural areas cannot easily travel outside of their community to access any services for various reasons. Therefore, LHWs provide direct healthcare services to them by traveling to their homes and are highly trusted by these women, as depicted by the formative findings. While it is true that LHWs have a burden on them, this study will be investigating the feasibility of introducing mental health-related services to their duties.<br><br>Moreover, these LHWs will be trained on the brief version of the GAD and PHQ tool which is just two questions, and this was chosen considering their relatively lower level of education and also limited time as highlighted by the reviewer. Additionally, the sessions included content on mental health awareness as a protective factor against any potential stigma.<br><br>The LHWs are just screening but they will be confirmed by the doctor at the primary health facility. Providing transport continuously is not sustainable in the long run and on a large scale. Also, in the LHW program model, the identified cases are routinely referred to the facility, where needed.<br><br>But we are providing transport to severe cases confirmed by the doctor at the health facility to a specialist, and this is specified on page numbers 9 and 12.
Background: Strongly focused on climate change and disaster context, but less emphasis on existing mental health service delivery challenges in Sindh (e.g., past PHC integration efforts). The cultural adaptation, translation, and validation tools, such as GAD-2/PHQ-2 for flood-affected rural women are not detailed.	Thank you for this comment. The background has been updated to include the mental health services in the rural areas of Pakistan. We have mentioned the translation of tools in the "Data Collection" section on page number 11.
The link between formative insights and the selected strategies seems weak – can you describe more about the integration of qualitative data into the intervention design?	Thank you, and we have now added further details, and these can be reviewed on page 8. We have mentioned in the formative phase, many WRA did not understand the term "mental health", hence the intervention included sessions on mental health awareness to increase their knowledge about common mental health disorders such as depression and anxiety. Moreover, stigma regarding mental health, for those who were aware, was challenged via mental health and resilience-building sessions and through counselling provided by healthcare facility staff at BHUs. WRA showed willingness for home-based mental health screenings and group sessions by LHWs, whom they trust, which were all incorporated in the intervention design.
Interestingly, there is no theory of change or a conceptual framework presented, which seems a key for such studies involving human behaviour and mental health.	Thank you for highlighting this, we have made the required addition. The conceptual framework utilized in this study is based off the ecological systems theory by Urie

Unfortunately, this limits the understanding of how screening, counselling, and resilience-building are expected to influence mental health outcomes.	Bronfenbrenner. This framework highlights the connection between an individual and their environment hence, fitting best with our multi-faceted research, incorporating different levels of influences.<br>A section termed "Conceptual Framework" has been added to the manuscript on page number 4.
If possible, the authors are requested to provide information on the costing of such a strategy or the cost-effectiveness benefit of this study.	We are collecting data regarding the cost, which is specified on page number 10.
The authors have discussed the distress protocol; however, operational details, including the timeline of action, delivery of more information, and referral tracking, appear to be lacking.	Operational details have been added to the section of "Data Collection" on page number 10.
The area and target population focused have a low level of literacy; therefore, written consent seems challenging. So, what are the alternatives?	Considering the low level of literacy amongst WRAs, data collectors acquired consent by reading out the consent form to the participants and then signatures or thumbprints were taken. Written consent was also for facility staff, LHWs and LHSs participating in the study. Please find this added in the section "Formative Phase" on page 6.

VERSION 2 - REVIEW

Reviewer 1
Name Azmat, Syed Khurram
Affiliation Jinnah Sindh Medical University, AAPNA - Institute of Public Health
Date 25-Aug-2025
COI

Thank you for addressing the comments.
